# A Systematic Review on the In Vivo Studies on Radiofrequency (100 kHz–300 GHz) Electromagnetic Field Exposure and Co-Carcinogenesis

**DOI:** 10.3390/ijerph21081020

**Published:** 2024-08-02

**Authors:** Rosanna Pinto, Lucia Ardoino, Paola Giardullo, Paola Villani, Carmela Marino

**Affiliations:** Division of Biotechnologies at ENEA, Italian National Agency for New Technologies, Energy, Environment and Sustainable Economic Development, Via Anguillarese, 301, 00123 Rome, Italy; lucia.ardoino@enea.it (L.A.); paola.giardullo@enea.it (P.G.); paola.villani@enea.it (P.V.); carmelamarino59@gmail.com (C.M.)

**Keywords:** electromagnetic fields, in vivo studies, co-carcinogenesis, tumor incidence, survival

## Abstract

In this systematic review, the potential role of in vivo RF–EMF exposure combined with the administration of well-known carcinogens in tumor promotion/progression is assessed. A total of 25 papers were included in the review. Each paper was assessed for Risk of Bias and for the attribution of the quality category. A meta-analysis was conducted on 18 studies, analyzing data for nine different organs/tumors to assess the potential increased risk for the onset of tumors as well as the effects on survival. A descriptive review was performed for the remaining seven eligible papers. In most cases, the results of the meta-analysis did not reveal a statistically significant difference in tumor onset between the sham and co-exposed samples. There was a numerically small increase in the risk of malignant tumors observed in the kidney and liver, as well as benign lung tumors. The level of evidence for health effects indicated “inadequate” evidence for an association between in vivo co-exposure to RF–EMF and known carcinogens and the onset of malignant or benign tumors in most of the analyzed tissues. Nevertheless, the limited number of eligible papers/studies for most of the analyzed tissues suggests that these results cannot be considered definitively conclusive.

## 1. Introduction

In recent decades, public concern regarding potential adverse health effects associated with exposure to radio frequency electromagnetic fields (RF–EMF) has grown. The increasing utilization of RF–EMF in various technologies has spurred numerous experimental research endeavors aimed at assessing the potential consequences of such exposure. In 2011, the International Agency for Research on Cancer (IARC) [1] classified RF–EMF as “possibly carcinogenic to humans” (Group 2B of its classification system) following an expert panel’s review of in vitro, in vivo and epidemiological studies. After the IARC’s classification, the large number of experimental and observational studies on this subject, required a systematic review to ensure a comprehensive evaluation. Recently, we conducted a systematic review [2] to investigate the effects of the in vivo exposure to RF–EMF (100 kHz–300 GHz) on carcinogenesis. The results highlighted the lack of a direct association between exposure to RF–EMF and an increased risk of cancer. The overall investigation yielded confidence ratings from “very low” to “moderate”, resulting in “inadequate” or “insufficient” evidence of health effects for a conclusive assessment of this association.

In this context, assessing the potential role of RF–EMF exposure in tumor promotion and/or progression is equally significant. It is known that certain agents, which directly cause alterations in the DNA molecule, act as initiators in the neoplastic process. On the other hand, some agents, while not inherently carcinogenic, can enhance the cancer-inducing effects of initiators, thereby promoting the development of neoplasms [3].

Beside the assessment of RF–EMF carcinogenic effects, several in vivo studies have been conducted aiming to investigate the effect of the combined exposure to cancer initiator/promoter agents and RF–EMF and to evaluate the possible mechanism underlying the process of tumor development. Consequently, the role of RF–EMF in co-promoting cancer was explored using well-characterized chemical/physical agents, known to induce specific neoplasms in different rodent strains. Like studies on RF–EMF carcinogenic effects, the frequencies used in mobile communication, in Wireless Fidelity (Wi-Fi) system and microwave ovens were the most widely investigated. Moreover, different exposure scenarios were applied in terms of dose of treatment, exposure modalities, duration, and daily timing.

The aim of this systematic review is to analyze the existing experimental studies involving animal exposure to RF–EMF in combination with known carcinogens. This analysis aims to evaluate the potential co-promotion/progression effects of RF–EMF exposure in terms of tumor incidence, latency, and survival in treated laboratory animals subjected to treatment.

## 2. Materials and Methods

The protocol of this systematic review is part of the comprehensive protocol titled Protocol for a systematic review of the in vivo studies on radiofrequency (100 kHz–300 GHz) electromagnetic fields exposure and cancer. This protocol was designed for the review of both carcinogenesis and co-carcinogenesis papers, registered on PROSPERO [CRD42020191105] and published in a peer-review journal [4]. The systematic review followed the guidelines and methodologies provided by the Cochrane Collaboration [5] and the Office of Health Assessment and Translation (NTP–OHAT) [6]. The Preferred Reporting Items for Systematic Reviews and Meta-Analyzes (PRISMA) [7] (PRISMA check list in Supplementary Material S1) was followed to draft this manuscript. However, it is important to note that the focus of the present paper is exclusively centered on the analysis of the combined exposure to RF–EMF and known carcinogens.

### 2.1. Eligibility Criteria

The review question was defined in terms of PECO (Population, Exposure, Comparison, Outcome):

- Population: rodents of both sexes, spanning all age groups and species, and encompassing various genetic backgrounds (including wild-type, transgenic, and tumor-prone animal models).
- Exposure: exposure to an electromagnetic field within the frequency range from 100 kHz to 300 GHz, with a precise characterization through dose assessment [8,9]. For frequencies up to 10 GHz, the assessment of the Specific Absorption Rate (SAR) was required. Any type of animal treatment with physical and chemical agents as tumor co-promoter was included.
- Comparison: the “sham” sample, i.e., animals treated with well-known carcinogens and kept under the same conditions as those used for irradiated animals but without RF–EMF exposure.
- Outcome: the onset of neoplasms in treated laboratory animals assessed in terms of tumor incidence (i.e., the number of animals developing cancer), latency (the time elapsed between treatment and the onset of neoplasms) and survival (number of live animals at the end of the experimental period).

While it was not explicitly specified in the protocol, papers that examined the role of RF–EMF exposure on animals implanted with tumor cells (“implanted tumors” or “xenografts”) were excluded from the analysis. This exclusion was based on the consideration that such studies do not qualify as co-carcinogenesis research, and their results cannot be readily compared with data obtained in chemical/physical co-carcinogenicity studies.

Additionally, papers exclusively reporting tumor-related parameters (i.e., genotoxicity, oxidative stress, etc.) were excluded from the analysis as well as papers that were not peer-reviewed or did not constitute original contributions (e.g., review articles, letters, and comments). There were no restrictions based on the year of publication, and only papers in the English language were included in the review.

### 2.2. Search Strategy

The search strategy for primary research papers was carried out on two database sources, PubMed and EMF Portal. It was integrated by including references from the selected papers and references from descriptive reviews on the same subject, which had been published over the years or conducted by international panels of experts [4]. This search strategy underwent a peer review as part of the protocol’s publication process.

For this specific review, an update using the same queries employed in Pinto et al. 2023 [2] was conducted on the aforementioned database sources. In addition, we extended our research to include the Scopus database.

### 2.3. Selection Process

The screening process involved two phases: an initial screening based on title and abstract, followed by full-text screening of the eligible articles, for final inclusion. The decision criteria were the same as those defined in [2,4]. In each phase, two reviewers (a biologist and a dosimetrist) independently assessed each article. Any discrepancies were resolved through discussion among the entire study team.

### 2.4. Data Extraction Form

The data extraction form, stored in an Excel file, was defined, and agreed upon before starting the full-text examination of eligible papers. The following information was extracted and organized into three separate sheets:

1. General information sheet on the experimental protocol:
  - Authors, publication year, title, journal,
  - Study design details, including the number of experimental and control groups, the number of animals per group, information on randomization, and blinding,
  - Characteristics of the animal model, such as species, strain, sex, genotype of animals (wild-type or transgenic),
  - Details of exposure duration (Long Term Exposure, longer than 52 weeks; Medium Term Exposure, longer than 9 weeks; Short Term Exposure),
  - Timing of treatment,
  - RF–EMF exposure details,
  - Type of well-known carcinogen agent, dosage administration,
  - Primary outcome(s), encompassing all tumor-related outcome measures. Numerical data were extracted from the text, tables, and figures (by using digital rulers where necessary). Notably, data related to animal survival were mainly derived from Kaplan–Meier curves, which were provided in most of the analyzed papers,
  - Methods employed to assess the endpoints,
  - Details on data analysis and the statistical evaluation process,
  - Information concerning animal deaths during the experimental period or instances where animals were euthanized due to suffering.
2. Results sheet where all raw data on tumor incidences, survival and latency were collected.
3. Risk of Bias (RoB) Sheet: this sheet also included a report on potential conflicts of interest present in all the included papers.

The primary objective of this initial data extraction scheme was to systematically arrange the information, enabling the subsequent evaluation of the Risk of Bias (RoB) for each individual paper. This information was also structured to create a summary table essentially serving as a database for meta-analysis. Within this summary table, each element corresponded to a treated/sham comparison (hereafter referred to as a ‘study’). Many of the papers reported experiments conducted with several treatment groups, each exposed to different RF–EMF levels and carcinogen doses. In the summary table, each article was listed as many times as the number of treated/sham comparisons, effectively representing the number of ‘studies’ conducted.

### 2.5. Risk of Bias (RoB) Evaluation 

For the evaluation of RoB and the assignment of quality categories, the same criteria applied in the assessment of the carcinogenic paper were followed [2,4,10]. A total of nine bias domains were considered:

1. Adequate randomization of administered dose or level of exposure,
2. Allocation of animals in treatment groups unknown to operators,
3. Evaluation of the experimental protocol,
4. Conducting treatment and analysis in a “blind” manner for animal groups (blind or double-blind),
5. Assessment of the exposure conditions,
6. The use of standardized and validated methods for determining the outcomes, and appropriate statistical methodologies,
7. Comprehensive reporting of all anticipated outcomes,
8. Calculation and justification of any losses of animals during the experimentation, whether due to death or for reasons other than those possibly foreseen by the experimental protocol,
9. The presence of any potential conflicts of interest.

To assign quality categories to the reviewed papers, each of these elements was evaluated and rated as follows: “++” indicated a definitely low risk of bias, “+” suggested a probably low risk of bias, “−” implied a probably high risk of bias, and “− −” signified a definitely high risk of bias. For item 9, a rating of “− −” was assigned to papers stemming from projects directly financed by telecommunication companies, while a rating of “−” was given to studies funded by consortia, including both public institutions and companies. 

Three quality categories were established based on the evaluation of these elements: “1” represented High Quality, “2” indicated Intermediate Quality, and “3” signified Low Quality. To determine the category particular emphasis was placed on RoB items 3 (adequacy of the experimental protocol), 5 (adequate dosimetry) and 6 (reliability of the methods used to evaluate the outcome). The quality category was only used for the assessment of confidence ratings and level of evidence for health effects.

### 2.6. Meta-Analysis: Strategy

The meta-analysis was conducted solely for organs/tumors that had data extracted from a minimum of 3 papers, regardless to the number of treated/sham comparisons [4]. Meta-Essentials tool (version 1.5) [11] was chosen and employed to perform the meta-analysis. This tool comprises a series of Excel workbooks, with one for each type of independent variable. It was developed by a team from the Rotterdam School of Management, Erasmus University, The Netherlands, under an ERIM Support Program and licensed under Creative Commons Attribution-Non-Commercial-ShareAlike 4.0 International [12].

The meta-analysis carried out is an Individual Participant Data (IPD) meta-analysis. All incidence data were presented in terms of the number of events and non-events in both the treated and control (sham) groups (2 × 2 table). These data were entered into the designed sheet (Input) of the Meta-Essentials and processed by the tool. The Risk Ratio (RR) was computed as the effect size measure [13]. The Random Effects model was chosen to calculate the absolute and relative weight of each treated/sham comparison [14,15,16]. Homogeneity/heterogeneity and the significance of the dataset were assessed through the evaluation of I2, tau, z-value, and *p*-value (with a significance level set at *p* < 0.01). Two summary tables were created for each organ, one for malignant and one for benign tumors, following the classification reported in Pinto et al. 2023 [2] to identify the tumor type. The results were reported in terms of the summary effect size, with the relative variability limits (lower and upper limit), significance and the Forest plot. 

The covariates “species” and “known carcinogen” were considered of interest for the subgroup analysis. Resulting summary effects and *p*-between values (indicating the significance of differences in variances among the subgroups) were extracted as summary information. 

The continuous variable SAR was used for the weighted regression analysis. The Meta-Essentials tool provides a scatter plot and a linear regression. The β moderator, the plot, the *p*-value and the R^2^ factor were extracted from this analysis.

### 2.7. Quality Assessment (Confidence Ratings and Level of Evidence for Health Effects)

The synthesis of evidence and the confidence rating in the body of evidence followed the guidelines set in [6,10], drawing on the guidance provided by the Grading of Recommendations Assessment, Development and Evaluation (GRADE) Working Group. For randomized in vivo studies, an initial rating of “high confidence” was assigned to each sample. Six domains were taken into account to potentially downgrade the quality of evidence: (i) limitations in the experimental design, (ii) RoB evaluation (excluding conflicts of interest), (iii) inconsistency, which considers the heterogeneity of the single sample, (iv) indirectness, which considers the study design’s ability (or lack thereof) to address the topic according to criteria of generality and transferability, (v) imprecision, which generally considers data as imprecise for ratio measures (e.g., RR) when the ratio of the upper to lower 95% CI for most studies is ≥10, (vi) publication bias. Two items were considered to upgrade the quality of evidence: (i) consistency between species and (ii) the presence of a clear dose response effect. Based on these considerations, the quality of evidence was classified into GRADE categories, which include “high”, “moderate”, “low”, or “very low”. Finally, the level of evidence for health effects was evaluated using the same tool.

## 3. Results

### 3.1. General Description of the Selected Studies

A total of 294 primary papers investigating the potential carcinogenic and/or co-carcinogenic effects of RF–EMF were selected (114 articles from EMF Portal, 112 from PubMed and 166 from other sources) and imported into EndNote with duplicates excluded.

Following an initial screening based on titles and abstracts, 237 papers were excluded, leaving 57 for full-text analysis. Subsequently, 11 papers were excluded for various reasons. In total, 46 papers were included in the systematic review with 23 papers focusing on carcinogenesis, 19 papers on co-carcinogenesis and 4 papers addressing both carcinogenesis and co-carcinogenesis. 

In this review a minor adjustment was made compared to previous systematic reviews on carcinogenic effects [2]: four papers related to both carcinogenicity and co-carcinogenicity, instead of five, were included. Indeed, in this review, data from the study by Tillmann et al. 2010 [17], initially classified only as a carcinogenesis article, were included. In fact, in this paper, despite the co-carcinogenesis study lacking a genuine sham control, the RF–EMF exposure modality (freely moving animals in the cages during the exposure) led to the consideration that the positive control for the co-carcinogenesis could be considered an appropriate comparator for the RF- EMF exposed group.

**Figure 1:**
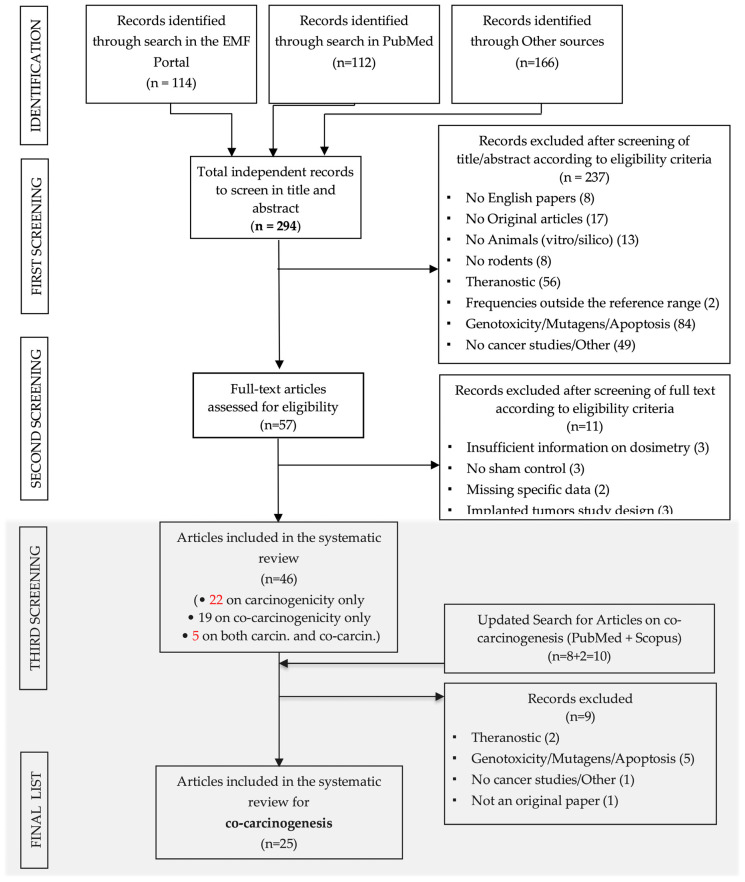
Final flow diagram of literature search: 25 papers were considered eligible for this systematic review. In red are highlighted the minor adjustments performed with respect [2].

Furthermore, an update using the same queries defined in Pinto et al. 2022 [4] was carried out on the previous databases as well as the Scopus database in April 2023. This updated search resulted in 10 new papers, of which 9 were excluded by the full-text analysis, and 1 was added to the list of eligible papers. As a result, 25 articles were included in this review. The flow chart illustrating the entire process of search and selection is presented in Figure 1.

The 25 co-carcinogenesis papers reported the results of 64 different treated/sham comparisons: 8 papers reported experiments conducted on a single treated/sham comparison [17,18,19,20,21,22,23,24], 7 papers examined 2 treated/sham comparisons [25,26,27,28,29,30,31], 4 papers included 3 treated/sham comparisons [32,33,34,35], 2 papers had 4 treated/sham comparisons [36,37], 2 papers explored 5 treated/sham comparisons [38,39], and finally, 2 papers presented 6 treated/sham comparisons [40,41]. A summary of the most relevant information related to the 64 treated/sham comparisons, including populations, exposure details and outcomes, is provided in Table 1.

Regarding the types of animals used in the selected papers (POPULATION) a total of 14 papers (32 treated/sham comparisons) described experiments performed on rats, while 11 papers (32 treated/sham comparisons) used mice. In all the studies, wild-type strains of rats and mice were employed, except for one paper (2 treated/sham comparisons) that used K2/ODC transgenic mice.

A total of 8 papers (14 treated/sham comparisons) conducted experiments on animals of both sexes, 11 papers (33 treated/sham comparisons) focused solely on female animals, and 6 papers (17 treated/sham comparisons) exclusively used males.

Regarding the characteristics of the electromagnetic signals (EXPOSURE), 20 papers (45 treated/sham comparisons) reported experiments involving exposure to mobile phone frequencies (800–900 MHz GSM, 800–900 MHz CDMA, 1700–1900 MHz DCS, 1700–2000 MHz UMTS/CDMA). Three papers (12 treated/sham comparisons) detailed experiments with exposures at 2450 MHz continuous wave (CW). One paper reported exposure to both 2450 MHz CW (1 treated/sham comparison) and 112 MHz (Amplitude Modulation at 16 Hz) (1 treated/sham comparison), while another paper (5 treated/sham comparisons) presented CW exposures to 94 GHz. Furthermore, 8 papers (18 treated/sham comparisons) described experiments involving localized exposures, while the remaining papers (17 articles and 46 treated/sham comparisons) concerned experiments with whole body exposures.

Regarding the dose (SAR), 5 papers (7 treated/sham comparisons) reported experiments performed with SAR values ≤ 0.1 W/kg, 19 papers (36 treated/sham comparisons) reported experiments with 0.1 < SAR ≤ 2 W/kg, 5 papers (11 treated/sham comparisons) reported experiments with 2 < SAR ≤ 6 W/kg, and finally, 3 papers (5 treated/sham comparisons) featured experiments with SAR values greater than 6 W/kg. One article (5 treated/sham comparisons) presented experiments at 94 GHz, exposing at power densities of 0.333 and 1 W/cm^2^. 

Most of the papers planned an exposure lasting longer than 4 months up 2 years, although the duration of exposure was often influenced by animal suffering/mortality caused by high doses of the carcinogen. It is worth noticing that, for this reason, the variable ‘duration’ was considered irrelevant for regression analysis.

Moreover, 18 papers (45 treated/sham comparisons) reported experiments with daily exposures lasting less than 5 h, 3 papers (7 treated/sham comparisons) reported experiments with daily 6 h exposures, 3 papers (7 treated/sham comparisons) detailed experiments with daily exposures exceeding 12 h and, finally, 1 paper (5 treated/sham comparisons) reported exposures of less than 1 min per day.

Regarding the co-carcinogens used in the studies:

- 7,12-dimethylbenz[a]anthracene (DMBA) was used in 8 papers (25 treated/sham comparisons), with one of these combining DMBA treatment with 12-O-tetradecanoyl phorbol-13-acetate (TPA),
- Ethylnitrosourea (ENU) was employed in 9 papers (17 treated/sham comparisons), where pregnant females were treated with a single ENU administration to assess the effects of RF–EMF on the development of tumors, especially in the central nervous system, induced by the transplacental transmission of the agent,
- Diethylnitrosamine (DEN) was used in 2 papers (2 treated/sham comparisons),
- 3,4-benzopyrene (BaP) was featured in 2 papers (11 treated/sham comparisons),
- 3-chloro-4-(dichloromethyl)-5-hydroxy-2(5H)-furanone (MX) was employed in 1 paper (2 treated/sham comparisons),
- Dimethylhydrazine (DMH) was used in 1 paper (1 treated/sham comparisons),
- X-rays were employed in 1 paper (2 treated/sham comparisons),
- UV radiation was used in 1 paper (4 treated/sham comparisons).

Regarding the site of the analyzed tumor: 4 papers (8 treated/sham comparisons) analyzed tumors in all tissues, the remaining papers focused on specific tumors: 7 papers (13 treated/sham comparisons) investigated brain tumors, 4 papers (15 treated/sham comparisons) studied breast cancer, 7 papers (25 treated/sham comparisons) examined skin cancer, 2 papers (2 treated/sham comparisons) assessed liver cancer and 1 paper (1 treated/sham comparisons) analyzed colon cancer. The choice of the target tissue depended on the specific carcinogenic agent used, as some are organ-specific (e.g., DMBA/TPA for skin).

Regarding the type of assessed OUTCOME, almost all papers (23) reported tumor incidence except for 2 papers reporting other outcome measures; 14 papers (37 treated/sham comparisons) also reported survival data. The latency was analyzed by 8 papers (27 treated/sham comparisons).

### 3.2. RoB of the Selected Papers

The results of the overall assessment of the RoB and the quality category of the co-carcinogenesis studies included in the analysis are presented in Table 2.

### 3.3. Incidence Analysis

The results of each eligible paper in terms of effect on tumor promotion/progression are shown in Table 3. All the raw data, including incidences, SAR, species, and carcinogen agents, are reported in Supplementary Material S2, for each organ/tumor.

Summary information on the potential increase in the risk of tumors onset in each organ/tissue, due to combined RF–EMF and co-carcinogen exposure, is reported in terms of RR in Table 4 for both malignant and benign tumors. The table includes the number of papers (column 2), the number of treated-sham comparisons (column 3), and the inclusion of the organ/tissue in the meta-analysis (column 4).

Results from the meta-analysis are presented from columns 5 to 11. As stated in the protocol, the meta-analysis was conducted only when a minimum of 3 papers per organ/tissue were selected resulting in the analysis of 18 papers for tumor incidence. The most commonly analyzed organs/tissues were brain (10 papers), skin (6 papers) and breast (5 papers).

**Figure 2:**
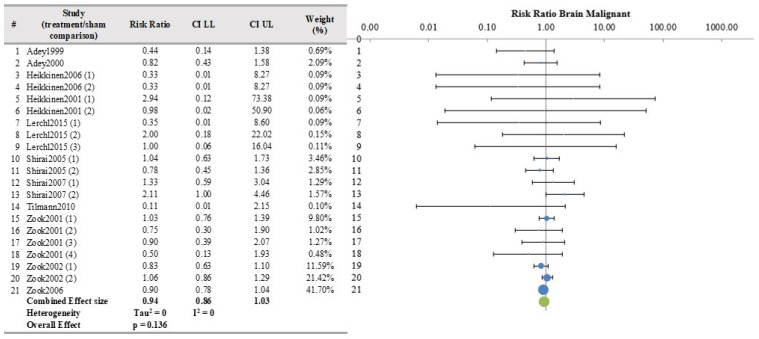
Meta-analysis results and relative forest plot for brain malignant tumors (blue points in the graph represent the RR of the singles studies, green representing the combined effect size, black bars representing the confidence interval limits reported in the table) [17,18,19,24,25,26,29,30,31,34,37].

**Figure 3:**
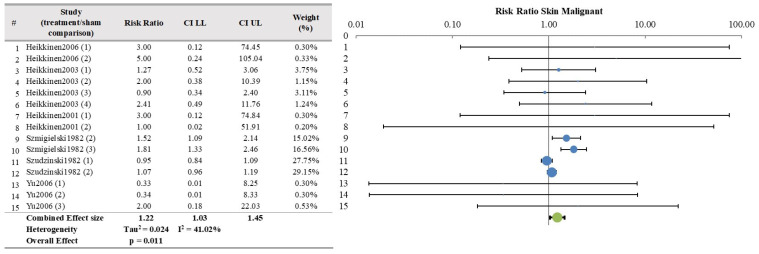
Meta-analysis results and relative forest plot for skin malignant tumors (blue points in the graph represent the RR of the singles studies, green representing the combined effect size, black bars representing the confidence interval limits reported in the table) [25,26,35,36,39,41].

**Figure 4:**
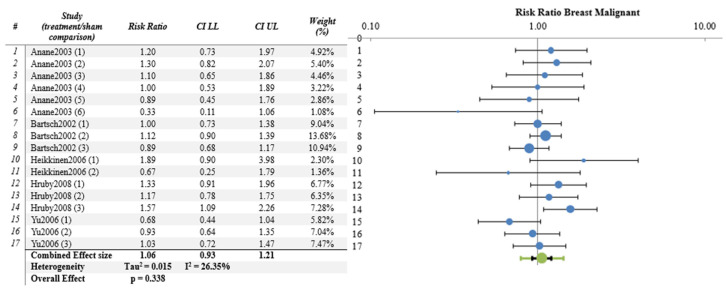
Meta-analysis results and relative forest plot for breast malignant tumors (blue points in the graph represent the RR of the singles studies, green representing the combined effect size, black bars representing the confidence interval limits reported in the table) [25,32,33,35,40].

**Figure 5:**
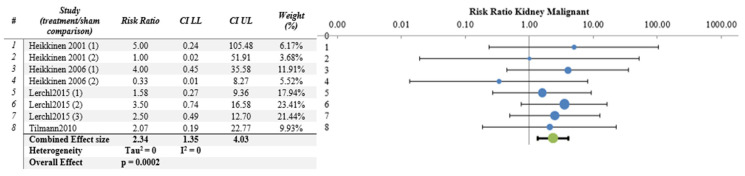
Meta-analysis results and relative forest plot for kidney malignant tumors (blue points in the graph represent the RR of the singles studies, green representing the combined effect size, black bars representing the confidence interval limits reported in the table) [17,25,26,34].

**Figure 6:**
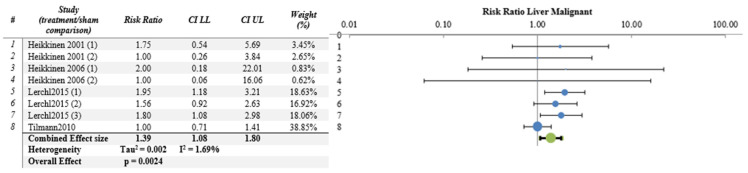
Meta-analysis results and relative forest plot for liver malignant tumors (blue points in the graph represent the RR of the singles studies, green representing the combined effect size, black bars representing the confidence interval limits reported in the table) [17,25,26,34].

The meta-analysis results were not significant for most organs/tissues as depicted in Figure 2, Figure 3 and Figure 4 and Supplementary Material S3. However, there were significant findings for malignant tumors of kidney (4 papers, 8 studies, RR = 2.34, CI 95%, 1.34–4.03, *p* = 0.0002) and liver (4 papers, 8 studies RR = 1.39, CI 95%, 1.08–1.80, *p* = 0.002) (Figure 5 and Figure 6 respectively), as well as for benign lung tumors (4 papers, 8 studies, RR = 1.65, CI 95%, 1.35–2.02, *p* = 4 × 10^−9^) (Table S3.27 Supplementary Material S3).

#### 3.3.1. Subgroup-Analysis

Subgroup analysis in most organs focused on the covariate “known carcinogen” and not species, as these two variables were often closely correlated, representing a standardized experimental animal model for studying the onset of a specific tumors. However, no statistical significance was found in any of the subgroup analyses for the covariate “known carcinogen” (see Supplementary Material S3).

For skin and brain, there is no univocal carcinogen-species reference system. Various carcinogens capable of inducing tumors in different species were identified for skin, leading to investigations with different carcinogenic animal models. In the case of brain tumors, no specific carcinogen has been validated, and different rodent species were treated.

However, in the brain sample, 19 out of 21 treatment/sham comparisons were treated with ENU, prompting a meta-analysis on this subset (Table S3.3 in Supplementary Material S3). Similarly, in the breast sample, 15 out of 17 treatment/sham comparisons were treated with DMBA, leading to a meta-analysis on this defined subset (Table S3.15 in Supplementary Material S3). In both cases, no statistically significant results were found.

The high correlation between animal species and the known carcinogen treatment allowed for the analysis of incidence data by species only for brain and skin samples. In these cases, carcinogen agents were not related to the species. Subgroup analysis by species for malignant tumors in the brain (21 treated/sham comparisons, Table S3.2 in Supplementary Material S3) and in skin (15 treated/sham comparisons, Table S3.17 in Supplementary Material S3) was conducted, but no significant differences were observed between species.

#### 3.3.2. Regression Analysis

Since the co-carcinogens investigated in the papers have very different characteristics and mechanisms of action, it is not proper to pool all the data together for the regression analysis on the variable SAR. For this reason, the regression analysis for SAR variable was only performed for subsets of samples from brain cancer animals treated with ENU (9 papers, Table S3.1 in Supplementary Material S3), and breast cancer animals treated with DMBA (4 papers, Table S3.2 in Supplementary Material S3). The results did not provide useful elements to define a dose–effect relationship in any of the analyzed samples.

#### 3.3.3. Quality Assessment (Confidence Ratings and Level of Evidence for Health Effects)

The evaluation of the quality of evidence for malignant and benign tumors followed a process starting from a “high quality” grade and considering the eight items for potential upgrades or downgrades, defined in the Methods section. The results of quality assessment are presented in Table 5.

For malignant tumors, a total of 9 organs/tumors were analyzed while 7 were considered for benign tumors to assess the confidence in the body of evidence regarding the effects of co-exposure to RF–EMF and known carcinogens.

All samples, except the brain for malignant tumors and skin for benign tumors, underwent a quality downgrade due to “some concern” limitations in the experimental design, primarily caused by a low number of animals in the sham groups (less than 50%). A similar downgrade was applied to the brain for both malignant and benign tumors as well as to skin for malignant tumors, primarily due the RoB being classified as “some concern”.

For all papers, there were “no concerns” related to inconsistency and indirectness, while “serious” imprecision was observed only for malignant tumors in the spleen (see Table S3.19 in Supplementary Material S3). It is worth noticing the difference in the downgrade assignment for skin malignant and benign tumors in the items “Design” and “RoB”. This difference was primarily due to two papers [39,41] with high RoB and “some concern” in the experimental design (as shown in Table 2), presenting incidence data for skin malignant tumors but not for skin benign tumors.

The lack of consistency and the absence of a dose–response relationship in all analyses precluded any upgrades in the quality assessment.

### 3.4. Survival Analyses

The survival data processed in this analysis represent the number of animals that were still alive at the end of the experimental period. The meta-analysis included 33 treated/sham comparisons and the combined effect size measure was expressed in terms of RR. The raw data for the survival analysis can be found in Table S2.34 in the Supplementary Materials S2.

**Figure 7:**
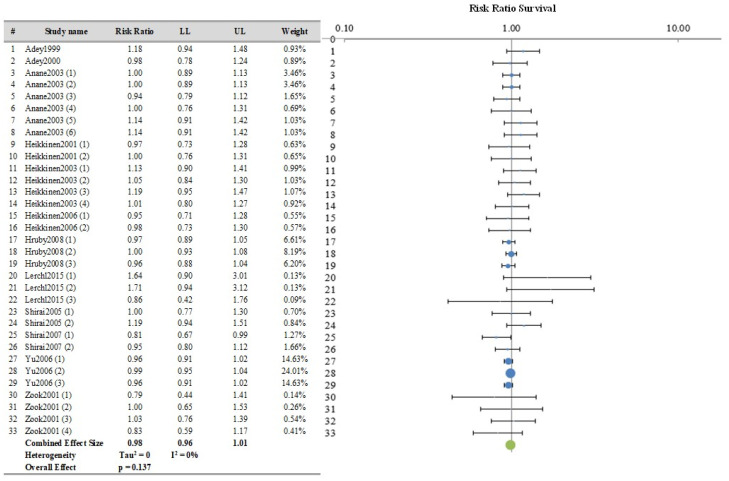
Meta-analysis results and relative forest plot for survival outcome measure (blue points in the graph represent the RR of the singles studies, green representing the combined effect size, black bars representing the confidence interval limits reported in the table) [18,19,25,26,29,30,33,34,35,36,37,40].

The results of meta-analysis, along with the Forest plot, are reported in Figure 7. The overall RR value was 0.98 (CI 95% 0.96–1.01). These results indicate that there was no statistically significant difference in survival between the sham and treated groups.

**Figure 8:**
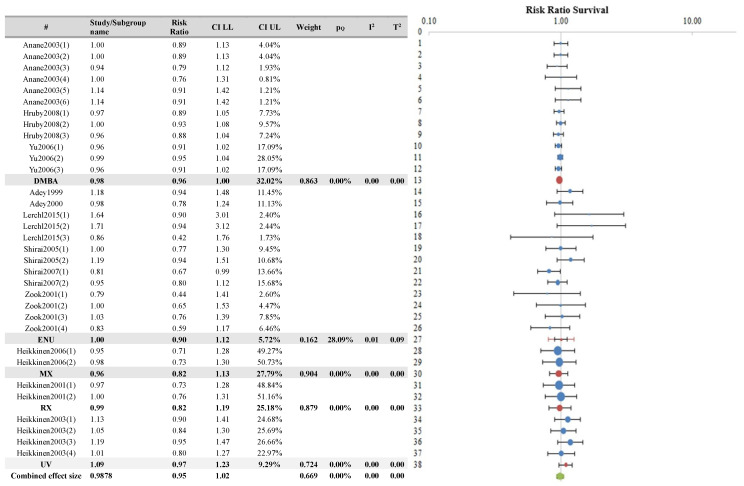
Survival outcome measure: subgroup analysis according to the covariate co-carcinogen agent (blue points in the graph represent the RR of the singles studies, red points represent the RR of the single covariates, green represents the combined effect size, and black bars represent the confidence interval limits reported in the table) [18,19,25,26,29,30,33,34,35,36,37,40].

**Figure 9:**
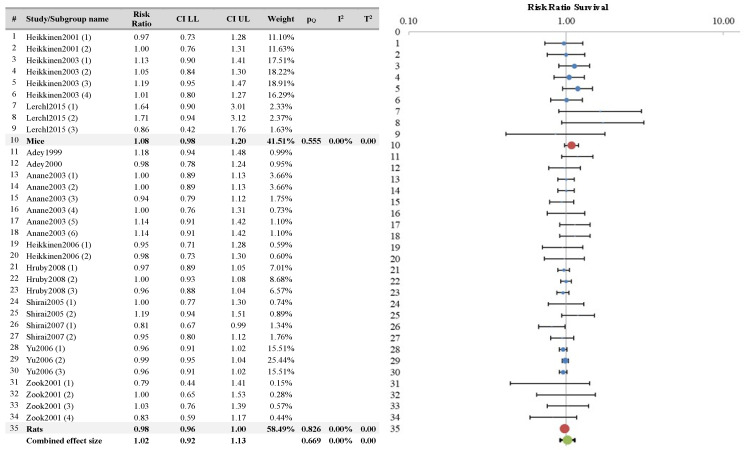
Survival outcome measure: subgroup analysis according to the covariate species (blue points in the graph represent the RR of the singles studies, red points represent the RR of the single covariates, green represents the combined effect size, and black bars represent the confidence interval limits reported in the table) [18,19,25,26,29,30,33,34,35,36,37,40].

Subgroup analyses were conducted based on the covariates “known carcinogen” and “species”, and the results are displayed in Figure 8 and Figure 9, respectively. These subgroup analyses too did not reveal any statistically significant differences among the groups.

Additionally, a regression analysis based on the SAR variable did not indicate a dose–effect response.

#### Quality Assessment (Confidence Ratings and Level of Evidence for Health Effects) for Survival

Survival data by 12 papers involving 33 studies (comprising 2109 animal exposed to RF–EMF and 1085 sham-exposed animals) were analyzed to assess the confidence in the body of evidence for the effects of co-exposure to RF–EMF and all the used carcinogens. An additional analysis was carried out to evaluate the body of evidence for individual carcinogens. The analysis followed the same criteria as described for tumor incidence. The results are summarized in Table 6, and it was found that there was “Evidence of no health effect” for all the analyses. This means that, with the available data, the combined evidence suggests no significant impact on the survival of animals due to the co-exposure to RF–EMF and the various carcinogens used in the studies.

### 3.5. Latency Analysis

The latency data provided by some papers represent the time interval (usually provided as the number of days) within which 50% of the animals developed tumors. Unfortunately, it was not possible to conduct an analysis of latency because only a few articles reported this outcome measure for all treatment groups. In some cases, only brief comments were provided to describe latency data while, in other papers, the latency outcome was reported with different metrics.

There was a total of 8 papers that included latency as an outcome measure. These covered 3 papers on breast cancer [32,35,40], 2 papers on skin cancer [39,41], 2 papers on brain tumors [24,31], and 1 paper considering all tumors [17]. The papers investigating breast and brain tumors reported no statistically significant differences in latency between the sham and treated groups. However, the two papers that investigated latency in the development of skin cancer after co-exposure to RF–EMF and benzopyrene reported a statistically significant acceleration of tumor growth in the treated groups. It is important to note that these papers were classified as “very low quality” for Risk of Bias (RoB) (see Table 2) due to the lack of information in the experimental protocol and data presentation, so these results may not be entirely reliable.

### 3.6. Qualitative Summary of the Excluded Works from the Meta-Analysis

Four papers were excluded from the meta-analysis due to substantial differences in experimental design or difficulties in data management. Here is a summary of their findings:

- Imaida et al. 1998a [22] and Imaida et al. 1998b [21]: these papers investigated the promotion role of RF–EMF exposure to 929.2 MHz or 1439 GHz, respectively, in rats treated with DEN. The animals received a single dose of DEN (200 mg/kg) and, after two weeks, they were exposed 90 min/day, 5 days/week, for 6 weeks to RF–EMF. After treatment, all animals were subjected to a partial hepatectomy, and the co-carcinogenic potential of the co-exposure was assessed analyzing the glutathione S-transferase placental form (GST-P) positive foci induction in the livers. The results indicated that the exposure to 929.2 MHz, as well as to 1.439 GHz RF–EMF, has no promoting effect on rat liver carcinogenesis. It was decided not to include the results of these articles in the meta-analysis due to the experimental design which, having foreseen the partial hepatectomy, made the data non-comparable with those reported by the other included papers. Furthermore, the tumor onset was not evaluated in terms of incidence, survival, or latency.
- Mason et al. 2001 [38]: This paper investigated the effects of single or repeated (2 exposures/week for 12 weeks) exposure to 94 GHz RF–EMF combined with DMBA or DMBA + TPA on mice skin. The authors reported the incidence data of skin tumors only through graphs. The results showed that, in any case, RF–EMF exposure did not promote or co-promote papilloma development. Due to the very high incidence of tumors in the positive control (TPA treatment), it was impossible to extrapolate neoplasm incidence numerical data related to sham and co-exposed samples.
- Wu et al. 1994 [23]: This paper investigated the effects of the combined exposure to RF–EMF and dimethylhydrazine (DMH) to assay the onset of colon tumor. Mice were treated with DMH (as tumor initiator) once per week for 14 weeks and with TPA (as a tumor promoter) once per week for 10 weeks beginning 3 weeks after the initial treatment with DMH. The animals were irradiated dorsally with RF–EMF 2.45 GHz for 3 h daily, 6 days per week, over a period of 5 months. The authors report the lack of tumor onset in both sham and treated samples. Because colon cancer was not assessed in any of the other papers included in this review, data were not included in the meta-analysis.

Three other papers were not included in meta-analysis because they reported zero tumor incidences in all treatment groups except for the positive controls, if present:

- Imaida et al. 2001 [20]: this paper assessed the effects of the co-exposure to DMBA, TPA and 1.5 GHz RF–EMF on mouse skin. Animals were treated with a topical application of DMBA on pre-shaved dorsal skin and divided in three groups: one group was exposed to 1.5 GHz RF–EMF 90 min a day, 5 days a week for 19 weeks, one group was placed in the exposure system without exposure, and the third group, the positive control, was weekly treated with TPA, a known tumor promoter in DMBA-induced skin carcinogenesis. The presence of tumors was only observed in the positive control sample, while the onset of tumors was not observed either in the DMBA sham control and in the animals treated with DMBA and RF–EMF.
- Paulraj et al. 2011 [28]: This paper investigated the co-carcinogenic effect of the exposure to 112 MHz or 2.45 GHz RF–EMF 2 h/day, 3 days a week for 16 weeks and a single dose of DMBA on mice skin. There was no tumor development in mice exposed to DMBA, as well as to DMBA and RF–EMF.
- Huang et al. 2005 [27]: this paper assessed the effects of the co-exposure to DMBA (a single administration), TPA and 849 or 1763 MHz RF–EMF on mouse skin. RF–EMF exposure was conducted for 2 cycles of 45 min exposure with a 15 min interval each day, 5 days a week for 19 weeks. There was no evidence of tumor onset either in the animals treated with DMBA or in the mice treated with DMBA and RF–EMF.

## 4. Discussion

In this work, we aimed to consolidate the existing knowledge regarding the potential impact of in vivo RF–EMF exposure, spanning the frequency range of 100 kHz to 300 GHz, on tumor promotion and progression combined with treatment with well-characterized chemical and physical carcinogens.

For this purpose, we conducted a systematic review analyzing the experimental data extracted from 25 papers, which were deemed eligible based on the criteria outlined in the protocol [4] and briefly summarized in the Methods section. For each paper, the RoB was assessed and its quality category determined. A quantitative analysis was performed on data extracted from 18 papers to address the potential increase in the risk of the tumor onset in animals exposed to known carcinogens combined with RF–EMF. The remaining seven papers underwent a qualitative analysis. Animal survival was also investigated. The RR was defined as the outcome measure for both tumor incidence and survival.

The results of most meta-analyses did not yield statistically significant findings. Notably, statistically significant RRs > 1 were observed only for the incidence of malignant kidney tumors (RR = 2.34, CI 95%, 1.34–4.03, *p* = 0.0002), malignant liver tumors (RR = 1.39, CI 95%, 1.08–1.80, *p* = 0.002) and benign lung tumors (RR = 1.65, CI 95%, 1.35–2.02, *p* = 4 × 10^−9^). It is worth mentioning that the increased incidence of tumors in the liver (malignant tumors) and lung (benign tumors) can be largely attributed to data from the papers by Tillmann et al. 2010 [17] and Lerchl et al. 2015 [34], the latter being a partial replication study of the former. These papers involved the administration of a single dose of ENU to pregnant mice followed by RF–EMF exposure during pregnancy. Additionally, the offspring continued to be exposed to RF–EMF throughout their lifespans, and the effects of RF–EMF/ENU co-exposure were assessed in various organs. It is important to note that these papers, among those using ENU, were the only ones to find a statistically significant difference in tumor incidence between sham and treated groups, albeit in specific organs (lung and liver). However, these findings did not extend to differences in mice survival. As for malignant kidney tumors, none of the included papers demonstrated statistically significant differences in tumor incidence between sham and treated groups (see Table 3). Nonetheless, when data from multiple papers and various co-carcinogens were analyzed together, the meta-analysis results indicated significant RRs > 1 (Figure 5). Regardless, the assessment of the body of evidence, using the GRADE approach, ascribed a “moderate” quality of evidence to the results obtained for malignant liver and kidney tumors and for benign lung tumors, translating to “moderate” evidence for health effects (Table 5). However, the limited number of papers (four) and studies (eight) constituting the liver, kidney, and lung samples as well as the diversity of carcinogenic agents employed (three) make it challenging to draw definitive conclusions regarding their level of evidence for health effects.

Given the significance of assessing the impact of RF–EMF on brain tissue, which was the most frequently studied organ in this systematic review (11 papers, 21 exposed/sham comparisons for malignant tumors), itis important to note that the findings of this systematic review did not confirm the findings of the previous systematic review on in vivo carcinogenesis studies [2], where we reported a ‘low’ level of evidence for health effects for brain, related to the weak positivity of most of the exposed/sham comparisons (18 vs. 8). In this systematic review, the results on brain revealed no statistical significance in the result and an ‘inadequate’ level of evidence for health effects.

Skin tissue, another frequently studied organ (6 papers and 15 exposed/sham comparisons for malignant tumors), produced no significant results in the meta-analysis for both malignant and benign tumors, resulting in an “inadequate” level of evidence for health effects for malignant tumors and with “no evidence” for health effects pertaining to benign skin tumors. The result for benign skin tumors cannot be considered conclusive due to the limited numbers of papers (3) and studies (8), as well as the use of different carcinogens (3).

In the case of other organs, an “inadequate” level of evidence for health effects was determined concerning the association between in vivo co-exposure to RF–EMF and known carcinogens and malignant/benign tumor incidence.

The analysis of animal survival provided evidence of “no health effect”.

Unfortunately, conducting a latency analysis was hindered due to difficulties in standardizing outcome measures among the eligible papers.

It is noteworthy that almost all selected papers (23 out of 25) investigated the combined exposure of RF–EMF with chemical agents. Only two papers explored the combined effects of RF–EMF/UV and RF–EMF/X-Rays. Moreover, excluding the paper of Anane et al. [40], all the studies included in the meta-analysis had a medium/long-term exposure period (Table 1); this characteristic is typical of this type of experimental study and is not the result of a selection made by the reviewers.

One of the eligibility criteria for this systematic review was publication in the English language. Although this is a limitation to the exhaustiveness of the review, considering that this type of study requires significant human resources and financial investment, as well as adequate facilities and extended durations, it can be assumed that the results of most of these studies are published in international journals.

## 5. Conclusions

In this systematic review, an “inadequate” level of evidence for health effects for an association between in vivo co-exposure RF–EMF and known-carcinogens and tumor incidence was assessed in most of the analyzed tissues. Although a slightly increased risk for malignant tumors, numerically significant, was observed in the kidney and liver, as well as for benign tumors in the lung, the limited number of eligible papers (4) and the use of different carcinogens (3) do not establish a robust foundation for assessing a “moderate” level of evidence for health effects.

Furthermore, this systematic review reveals the scarcity of papers focusing on the combined exposure of RF–EMF and physical agents. It may be of interest to delve deeper into studies involving RF–EMF/UV combined exposure, especially in tissues like skin. The skin is particularly relevant as it represents one of the primary targets for millimeter wave exposure associated with the latest telecommunication signals (5G), due to their millimetric penetration depth.

In the future, it is conceivable that an updated version of this review will be warranted to provide the scientific community and decision-makers with current and relevant information on this issue.

## Figures and Tables

**Table 1:** Summary of data extracted from all the eligible papers.

ID Paper [Ref]	ID Study Number	Study (Treatment-Sham Comparison)	Species Animal Type Prone/Wild Type	No of Animals/Groups	Sex	Carcinogenic Agent (CA)	Dose of CA	Frequency (MHz) Modulation	SAR (W/Kg)	WbSAR/ Local SAR	Duration (w)	Timing (h/d, d/w)	Organ	Type of Tumor (Mal/Ben)	Outcome Measure	Note
1 [19]	1	Adey 1999	Rats F344 Ut WT	116	M + F	ENU	4 mg/kg at gestation day 18	836 TDMA	1–1.60	local	94	2 h/d 4 d/w	CNS/Brain	Malignant tumors	Incidence Survival	SAR values related to growth
2 [18]	2	Adey 2000	Rats F344 Ut WT	90	M + F	ENU	4 mg/kg at gestation day 18	836 TDMA	0.74–1.60	local	96	2 h/d 4 d/w	CNS/Brain	Malignant tumors	Incidence Survival	SAR values related to growth
3 [40]	3	Anane 2003 (1)	Rats Spraque-Dawley WT	16	F	DMBA	10 mg sigle dose	900 TDMA	1.4	wb	9	2 h/d 5 d/w	Breast	Malignant tumors	Incidence/latency Survival	
	4	Anane 2003 (2)		16 *					2.2							
	5	Anane 2003 (3)		16 *					3.5							
	6	Anane 2003 (4)		16					0.1							
	7	Anane 2003 (5)		16 *					0.7							
	8	Anane 2003 (6)		16 *					1.4							
4 [32]	9	Bartsch 2002 (1)	Rats Spraque-Dawley WT	20	F	DMBA	8.75 mg single dose	900 TDMA	0.0175–0.07	wb	Until all animals developed tumors	24 h/d 7 d/w	Breast	Malignant, Benign tumors	Incidence/Latency	
	10	Bartsch 2002 (2)		20												
	11	Bartsch 2002 (3)		20												
5 [26]	12	Heikkinen 2001 (1)	Mice CBA/S WT	50	F	RX	4 Gy total (three equal fractions of 1.33 Gy at 1-week intervals	902.5 CW	1.5	wb	78	1.5 h/d 5 d/w	All	Malignant, Benign tumors	Incidence Survival	
	13	Heikkinen 2001 (2)		50				902.5 TDMA	0.35							
6 [36]	14	Heikkinen 2003 (1)	Mice K2 ODC-transgenic	20	F	UV	1.2 Human MED 3 times a week	894 DAMPS	0.5	wb	52	1.5 h/d 5 d/w	Skin	Malignant, Benign tumors	Incidence Survival	
	15	Heikkinen 2003 (2)		26				894 DAMPS								
	16	Heikkinen 2003 (3)		21				902 TDMA								
	17	Heikkinen 2003 (4)		26				902 TDMA								
7 [25]	18	Heikkinen 2006 (1)	Rats Winstar WT	72	F	MX	1.7 mg/kg daily	900 TDMA	0.3 0.9	wb	104	2 h/d 5 d/w	All	Malignant, Benign tumors	Incidence Survival	
	19	Heikkinen 2006 (2)		72 *												
8 [33]	20	Hruby 2008 (1)	Rats Spraque-Dawley WT	100	F	DMBA	17 mg/kg single dose	902 TDMA	0.4 1.3 4	wb	27	4 h/d 5 d/w	Breast	Malignant, Benign tumors	Incidence Survival	
	21	Hruby 2008 (2)		100 *												
	22	Hruby 2008 (3)		100 *												
9 [27]	23	Huang 2005 (1)	Mice ICR WT	20	M	DMBA	100 mg single dose	894 CDMA	0.4	wb	19	1.5 h/d 5 d/w	Skin	Malignant, Benign tumors	Incidence	The incidence is 0 in all groups. The paper is not included in the meta-analysis
	24	Huang 2005 (2)		20 *				1763 CDMA								
10 [20]	25	Imaida 2001	Mice ICR WT	48	F	DMBA	100 mg single dose	1500 TDMA	0.084	wb	19	1.5 h/d 5 d/w	Skin	Malignant, Benign tumors	Incidence	SAR 2 W/kg skin near field exposure
11 [22]	26	Imaida 1998a	Rats F344 WT	92	M	DEN	200 mg/kg single dose	1500 TDMA	0.680–0.453	wb	6	1.5 h/d 5 d/w	Liver	GST-P Positive Liver Foci	Nor incidence or latency	SAR 1.91–0.937 W/kg in the liver near field exposure
12 [21]	27	Imaida 1998b	Rats F344 WT	96	M	DEN	200 mg/kg single dose	929.2 TDMA	0.80–0.58	wb	6	1.5 h/d 5 d/w	Liver	GST-P Positive Liver Foci	Nor incidence or latency	SAR 2–1.7 W/kg in the liver near field exposure
13 [34]	28	Lerchl 2015 (1)	Mice B6C3F1 hybrids (Ut) WT	96	F	ENU	40 mg/kg at gestation day 14	1966 CDMA	0.04 0.4 2	wb	72	24 h/d 7 d/w	All	Malignant, Benign tumors	Incidence Survival	
	29	Lerchl 2015 (2)		96 *												
	30	Lerchl 2015 (3)		96 *												
14 [38]	31	Mason 2001 (1)	Mice SENCAR WT	55	F	DMBA	10 nmol DMBA single dose	94 GHz CW	1 W/cm^2^	local	0	10 sec	Skin	Skin Papilloma	Incidence	Data from graph not readable
	32	Mason 2001 (2)		35		DMBA	10 nmol DMBA single dose		333 mW/cm^2^		12			Skin Papilloma	Incidence	Data from graph not readable
	33	Mason 2001 (3)		35		DMBA + TPA	10 nmol DMBA single dose 0.85 nmol TPA twice a week		333 mW/cm^2^		12			Skin Papilloma	Incidence	Data from graph not readable
	34	Mason 2001 (4)		35		DMBA	10 nmol DMBA single dose		333 mW/cm^2^		12			Not specified	Epidermal tickness	Nor incidence or latency
	35	Mason 2001 (5)		35		DMBA + TPA	10 nmol DMBA single dose 0.85 nmol TPA twice a week		333 mW/cm^2^		12			Not specified	Epidermal tickness	Nor incidence or latency
15 [28]	36	Paulraj 2010	Mice Swiss Albino WT	18	M	DMBA	100 mg single dose	112 AM 16 Hz	0.75	wb	16	2 h/d 3 d/w	Skin	Malignant tumors	Incidence	The incidence is 0 in all groups. The paper is not included in the meta-analysis
	37	Paulraj 2010		18				2450 CW	0.1		17					
16 [30]	38	Shirai 2005 (1)	Rats F344 Ut WT	100	M + F	ENU	4 mg/kg at gestation day 18	1439 TDMA	0.67	local (head)	104	1.5 h/d 5 d/w	CNS/Brain	Malignant, Benign tumors	Incidence/ Survival	wb SAR provided as lower than 0.4 W/kg always
	39	Shirai 2005 (2)		100 *					2							
17 [29]	40	Shirai 2007 (1)	Rats F344 Ut WT	100	M + F	ENU	4 mg/kg at gestation day 18	1950 CDMA	0.67 2	local (head)	104	1.5 h/d 5 d/w	CNS/Brain	Malignant, Benign tumors	Incidence/ Survival	wb SAR provided as lower than 0.4 W/kg always
	41	Shirai 2007 (2)		100 *												
18 [41]	42	Szmigielski 1982 (1)	Mice Balb/c WT	40	M	BaP	0.01 mL of 5% 3.4 benzopyrene every 2nd day of the week, over 5 months, starting 1 month after MW exposure	2450 CW	2–3	wb	13	2 h/d 6 d/w	Skin	Malignant, Benign tumors	Latency	The study aim is the latency; incidendence data are only provided for groups 5 and 6 (included in the meta-analysis)
	43	Szmigielski 1982 (2)		40					2–3		13				Latency	
	44	Szmigielski 1982 (3)		40 *					6–8		13				Latency	
	45	Szmigielski 1982 (4)		40 *					6–8		13				Latency	
	46	Szmigielski 1982 (5)		40					2–3		22				Incidence/latency	
	47	Szmigielski 1982 (6)		40 *					6–8		22				Incidence/latency	
19 [39]	48	Szudzinski 1982 (1)	Mice Balb/c WT	100	M	BaP	0.01 mL of 5% 3.4 benzopyrene every 2nd day of the week, over 6 months simultaneously MW exposure	2450 CW	2	wb	27	2 h/d 6 d/w	Skin	Malignant, Benign tumors	Incidence/latency	The study aim is the latency; It has been possible to extrapolate the incidence, at the end of the observatoion period, only for groups 1 and 2.
	49	Szudzinski 1982 (2)		100 *					6		27				Incidence/latency	
	50	Szudzinski 1982 (3)		100					4		4				Latency	
	51	Szudzinski 1982 (4)		100					4		9				Latency	
	52	Szudzinski 1982 (5)		100 *					4		13				Latency	
20 [17]	53	Tillman 2010	Mice B6C3F1 hybrids (Ut) WT	60	F	ENU	40 mg/kg at 14th day of pregnancy	1966 CDMA	2.1–5.5	wb	75	20 h/d 7 d/w	All tumors	Malignant, Benign tumors	Incidence/latency	
21 [23]	54	Wu 1994	Mice Balb WT	54	M + F	DMH	15 mg/kg once a week for 14 weeks 20 mg/kg once a week for next 8 weeks	2450 CW	10–12	wb	22	3 h/d 6 d/w	Colon	Malignant tumors	Incidence	The paper is the only one focused on the Colon and it doesn’t provide any other data
22 [35]	55	Yu 2006 (1)	Rats Spraque-Dawley WT	100	F	DMBA	35 mg/kg single dose	900 TDMA	0.44	wb	26	4 h/d 5 d/w	Breast	Malignant, Benign tumors	Incidence/latency Survival	This paper is not included in the meta-analysis of benign tumors. Benign tumors number is underestimated because rats with carcinoma and benign tumors are counted only in carcinomas group
	56	Yu 2006 (2)		100					1.33							
	57	Yu 2006 (3)		100 *					4							
23 [37]	58	Zook 2001 (1)	Rats Spraque-Dawley (Ut) WT	60	M + F	ENU	10 mg/kg at 15th day of pregnancy	860 PRF	1	local (head)	56 ^#^	6 h/d 5 d/w	CNS/Brain	Malignant tumors	Incidence/ Survival	^#^ Animals are sacrificed at 394 days because 75% and over were found death
	59	Zook 2001 (2)		60			2.5 mg/kg at 15th day of pregnancy				95					Data from the paper are used only for brain tumor because other data requires a pre-processing process that is too complex and with many factors of uncertainty
	60	Zook 2001 (3)		60							95					
	61	Zook 2001 (4)		120				860 CW			95					
24 [31]	62	Zook 2002 (1)	Rats Spraque-Dawley (Ut) WT	180	M + F	ENU	6.3 mg/kg at 15th day of pregnancy	860 PRF	1	local (head)	39	6 h/d 5 d/w	CNS/Brain	Malignant tumors	Incidence/latency	
	63	Zook 2002 (2)					10 mg/kg at 15th day of pregnancy				39					
25 [24]	65	Zook 2006	Rats Spraque-Dawley (Ut) WT	360	M + F	ENU	6.3 and 10 mg/kg at 15th day of pregnancy	860 PRF	1	local (head)	39	6 h/d 5 d/w	CNS/Brain	Malignant tumors	Incidence/latency	ENU doses pooled; data on survival were not used because they were inconsistent with other data

Column 5: * close to the number of animals /groups means that the sham group is shared between several treated groups; Column 6: Information about sex is reported bau; Column 7: All the carcinogen agents are reported with their acronymous (ENU: Ethylnitrosurea, DMBA: Dimethylbenz(a)anthracene, RX: X Rays, UV: UltraViolet Radiation, MX: 3-Chloro-4-(dichloromethyl)-5-hydroxy-2(5H)-furanone, TPA: Tetradecanoyl phorbol acetate, BaP: Benzo[a]pyrene, DMH: Dimethylhydrazine); Column 8: The doses of the carcinogens agents are reported as provided by the authors, so they can result in a non-uniform format.

**Table 2:** RoB evaluation of all the eligible papers.

Paper	Item Score (- -, -, +, ++)									Quality Category (1–3)
	1	2	3	4	5	6	7	8	9	
Adey 1999 [18]	+	+	+	+	-	++	++	++	-	2
Adey 2000 [19]	+	+	+	+	-	++	++	++	-	2
Anane 2003 [40]	+	+	+	++	++	++	- -	+	+	1
Bartsch 2002 [32]	+	+	+	++	++	+	++	++	- -	2
Heikkinen 2006 [25]	++	+	++	++	++	++	++	++	-	1
Heikkinen 2003[36]	++	++	++	+	++	++	++	++	-	1
Heikkinen 2001 [26]	++	+	++	++	++	++	++	++	-	1
Hruby 2008 [33]	++	++	++	++	++	++	++	++	+	1
Huang 2005 [27]	+	+	+	-	+	+	++	++	++	2
Imaida 2001 [20]	+	+	+	-	-	-	++	-	- -	3
Imaida 1998 a [22]	+	+	+	-	-	-	++	-	- -	3
Imaida 1998 b [21]	+	+	+	-	-	-	++	-	- -	3
Lerchl 2015 [34]	++	++	++	++	++	++	++	++	++	1
Mason 2001 [38]	++	+	++	++	++	++	+	+	++	2
Paulraj 2010 [28]	+	-	+	-	+	+	-	++	+	2
Shirai 2005 [30]	++	++	++	++	++	++	+	++	++	1
Shirai 2007 [29]	++	++	++	++	++	++	+	++	++	1
Szimigielski 1982 [41]	- -	- -	++	- -	+	++	-	-	++	3
Szudzinski 1982 [39]	- -	- -	++	- -	+	++	-	++	++	3
Tillmann 2010 [17]	++	++	-	++	++	++	++	++	++	2
Wu 1994 [23]	++	- -	+	- -	+	++	++	++	++	2
Yu 2006 [35]	++	++	++	++	++	++	++	++	- -	1
Zook 2006 [24]	++	- -	+	++	++	++	- -	++	- -	2
Zook 2002 [31]	++	- -	+	++	++	++	++	++	- -	1
Zook 2001 [37]	++	- -	+	++	++	++	- -	++	- -	2
1. Randomized exposure level;										
2. Allocation concealment of study groups;										
3. Evaluation in the study design or analysis of possible important confounding and modifying variables;										
4. Blinding of research personnel;										
5. Confidence in the exposure characterization (dosimetry);										
6. Confidence in the outcome assessment;										
7. All measured outcomes reported;										
8. Attrition/exclusion rate;										
9. Possible conflicts of interest: “- -” was assigned to papers stemming from projects directly financed by telecommunication companies, while a rating of “-” was given to studies funded by consortia including both public institutions and companies.										

**Table 3:** Summary of evidence in each eligible paper.

Papers	Inclusion in MA	Presence of Effects
Adey 2000 [18]	Yes	No effects
Adey 1999 [19]	Yes	No effects
Anane 2003 [40]	Yes	No effects
Bartsch 2002 [32]	Yes	No effects
Heikkinen 2006 [25]	Yes	No effects
Heikkinen 2003 [36]	Yes	No effects
Heikkinen 2001 [26]	Yes	No effects
Hruby 2008 [33]	Yes	Significantly more animals with malignant breast neoplasms and significantly more animals with adenocarcinoma in the high-dose group than in sham-exposed group Significantly fewer animals with benign neoplasms in the RF-exposed groups than in the sham-exposed group
Huang 2005 [27]	No	No effects
Imaida 2001 [20]	No	No effects
Imaida 1998a [22]	No	No effects
Imaida 1998b [21]	No	No effects
Lerchl 2015 [34]	Yes	Significantly higher numbers of tumors of the lungs and livers in exposed animals than in sham-exposed controls Significantly higher numbers of lymphomas in exposed animals than in sham-exposed controls A clear dose-response effect is absent
Mason 2001 [38]	No	No effects
Paularj 2010 [28]	No	No effects
Shirai 2007 [29]	Yes	No effects
Shirai 2005 [30]	Yes	No effects
Szmigielski 1982 [41]	Yes	Acceleration of cancer development
Szudzinski 1982 [39]	Yes	Acceleration of cancer development
Tillmann 2010 [17]	Yes	Significantly more animals with lung carcinoma in exposed group than in sham-exposed group Significantly more animals with liver adenoma in exposed group than in sham-exposed group
Wu 1994 [23]	No	No effects
Yu 2006 [35]	Yes	No effects
Zook 2006 [24]	Yes	No effects
Zook 2002 [31]	Yes	No effects
Zook 2001 [37]	Yes	No effects

**Table 4:** Summary of meta-analysis results for malignant and benign tumors.

Organ/Tumor	Number of Papers	Number of Comparisons Exposed/Sham	Meta-Analysis	Risk Ratio	LL	UL	Two Tailed *p*-Value	Tau^2^	I^2^ (%)	z-Value
Malignant Tumors										
Adrenals	1	2	NO							
Brain	11	21	YES	0.939	0.860	1.025	0.1356	0	0	−1.49
Female Genital system	2	4	NO							
Heart	1	2	NO							
Histiocytic Sarcoma	4	8	YES	0.749	0.401	1.398	0.2730	0	0	−1.10
Hypophysis	1	2	NO							
Kidney	4	8	YES	2.335	1.352	4.033	0.0002	0	0	3.67
Liver	4	8	YES	1.392	1.075	1.802	0.0024	0.0020	1.70	3.03
Lung	4	8	YES	1.057	0.912	1.224	0.3749	0.0110	48.80	0.89
Lymphoma	4	8	YES	1.302	0.873	1.941	0.1189	0.0130	5.40	1.56
Breast	5	17	YES	1.062	0.931	1.210	0.3377	0.0150	26.40	0.96
Mesenteric lymph node	1	2	NO							
Pancreas	1	2	NO							
Sensor organs (Harderian gl.)	1	2	NO							
Skin	6	15	YES	1.224	1.031	1.452	0.0116	0.0240	41.00	2.53
Spleen	3	6	YES	0.589	0.123	2.810	0.3849	0.0018	0.08	−0.87
Thymus	1	2	NO							
Benign Tumors										
Adrenals	1	2	NO							
Brain	5	10	YES	0.537	0.242	1.192	0.0776	0	0	−1.76
Female Genital system	2	4	NO							
Heart	1	2	NO							
Hypophysis	1	2	NO							
Kidney	4	8	YES	0.845	0.472	1.512	0.4934	0	0	−0.68
Liver	4	8	YES	1.045	0.787	1.388	0.7137	0.058	50.2	0.37
Lung	4	8	YES	1.651	1.351	2.017	4 × 10^−9^	0	0	5.91
Breast	3	8	YES	0.887	0.678	1.160	0.2905	0.036	52.3	−1.06
Mesenteric lymph node	1	2	NO							
Pancreas	1	2	NO							
Sensor organs (Harderian gl.)	1	2	NO							
Skin	3	8	YES	0.644	0.395	1.050	0.0333	0	0	−2.13
Spleen	3	6	YES	1.030	0.419	2.528	0.9338	0	0	0.08
Thymus	1	2	NO							
Thyroid	1	2	NO							

**Table 5:** Quality and Health Evidence of malignant and benign tumor analysis.

	Studies (Groups/Papers)	Design	RoB	Inconsistency	Imprecision	Publication Bias	Total Exposed Animals	Total Sham Animals	Relative Effect RR (CI 95%)	Quality of Evidence	Health Evidence
Malignant Tumors											
Brain	21/11	No concern	Some concern (−1)	No (I^2^ = 0)	No serious	No	2096	1585	0.94 (0.86–1.03)	Moderate	Inadequate
Histiocytic sarcoma	8/4	Some concern (−1)	No concern	No (I^2^ = 0)	No serious	No	587	278	0.75 (0.40–1.40)	Moderate	Inadequate
Kidney	8/4	Some concern (−1)	No concern	No (I^2^ = 0)	No serious	No	585	278	2.34 (1.34–4.03)	Moderate	Moderate
Liver	8/4	Some concern (−1)	No concern	No (I^2^ = 1.7)	No serious	No	586	278	1.39 (1.08–1.80)	Moderate	Moderate
Lung	8/4	Some concern (−1)	No concern	No (I^2^ = 48.8)	No serious	No	587	278	1.06 (0.91–1.22)	Moderate	Inadequate
Lymphoma	8/4	Some concern (−1)	No concern	No (I^2^ = 5.4)	No serious	No	587	278	1.30 (0.87–1.94)	Moderate	Inadequate
Breast	8/4	Some concern (−1)	No concern	No (I^2^ = 26.4)	No serious	No	899	364	1.06 (0.93–1.21)	Moderate	Inadequate
Skin	15/6	Some concern (−1)	Some concern (−1)	No (I^2^ = 41)	No serious	No	917	452	1.22 (1.03–1.45)	Low	Inadequate
Spleen	6/3	Some concern (−1)	No concern	No (I^2^ = 0.08)	Seriuos (-1)	No	487	228	0.59 (0.12–2.81)	Low	Inadequate
Benign Tumors											
Brain	10/5	Some concern (−1)	Some concern (−1)	No (I^2^ = 0)	No serious	No	886	428	0.54 (0.24–1.19)	Low	Inadequate
Kidney	8/4	Some concern (−1)	No concern	No (I^2^ = 0)	No serious	No	585	278	0.84 (0.47–1.51)	Moderate	Inadequate
Liver	8/4	Some concern (−1)	No concern	Yes (−1) (I^2^ = 50.2)	No serious	No	586	278	1.05 (0.79–1.39)	Low	Inadequate
Lung	8/4	Some concern (−1)	No concern	No (I^2^ = 0)	No serious	No	587	278	1.65 (1.35–2.02)	Moderate	Moderate
Breast	8/4	Some concern (−1)	No concern	Yes (−1) (I^2^ = 52.3)	No serious	No	504	232	0.89 (0.68–1.16)	Low	Inadequate
Skin	8/3	No concern	No concern	No (I^2^ = 0)	No serious	No	338	212	0.64 (0.39–1.05)	High	Evidence of no health effect
Spleen	6/3	Some concern (−1)	No concern	No (I^2^ = 0)	No serious	No	487	228	1.03 (0.42–2.53)	Moderate	Inadequate

Design: Some concern (−1) when the number of sham animals is less than 50% of the exposed animals. RoB: Some concern: some studies show “−” in some relevant items; Conflict of interest item is not considered. Inconsistency: No if I^2^ < 50%, Yes (-1) I^2^ > 50% (up to 75%). Imprecision: Data are generally considered imprecise for ratio measures (e.g., RR) when the ratio of the upper to lower 95% CI for most studies is ≥10.

**Table 6:** Quality and Health Evidence of survival analysis.

	Studies (Groups/Papers)	Design	RoB	Inconsistency	Imprecision	Publication Bias	Total Exposed Animals	Total Sham Animals	Relative Effect RR (CI 95%)	Quality of Evidence	Health Evidence
Survival	33/12	No concern	No concern	No (I^2^ = 0)	No serious	No	2109	1085	0.98 (0.96–1.01)	High	Evidence no health effect
Survival with DMBA	12/3	No concern	No concern	No (I^2^ = 0)	No serious	No	696	332	0.98 (0.96–1.00)	High	Evidence no health effect
Survival with ENU	13/6	No concern	No concern	No (I^2^ = 28)	No serious	No	1074	684	1.00 (0.90–1.12)	High	Evidence no health effect
Survival with MX	2/1	No concern	No concern	No (I^2^ = 0)	No serious	No	144	72	0.96 (0.82–1.13)	High	Evidence no health effect
Survival with RX	2/1	No concern	No concern	No (I^2^ = 0)	No serious	No	100	50	0.99 (0.82–1.19)	High	Evidence no health effect
Survival with UV	4/1	No concern	No concern	No (I^2^ = 0)	No serious	No	95	45	1.09 (0.97–1.23)	High	Evidence no health effect

Design: Some concern (−1) when the number of sham animals is less than 50% of the exposed animals; RoB: Some Concern: some studies show “−” in some relevant items; Conflict of interest item is not considered; Inconsistency: No if I^2^ < 50%, Yes (-1) I^2^ > 50% (up to 75%); Imprecision: Data are generally considered imprecise for ratio measures (e.g., RR) when the ratio of the upper to lower 95% CI for most studies is ≥10.

## Data Availability

Authors have access to all scientific databases to collect all relevant papers for the systematic review.

## Supplementary Materials

The following supporting information can be downloaded at: https://www.mdpi.com/article/10.3390/ijerph21081020/s1, Supplementary Material 1 (S1): Prisma Check List; Supplementary Material 2 (S2): Incidence raw data extracted by eligible papers; Supplementary Material 3 (S3): Results of meta-analysis for all organs.
