# Supplementary material for: A Systematic Review on the In Vivo Studies on Radiofrequency (100 kHz–300 GHz) Electromagnetic Field Exposure and Co-Carcinogenesis
Source: Int J Environ Res Public Health. 2024 Aug 2;21(8):1020. doi: 10.3390/ijerph21081020 (PMC11354106; doi:10.3390/ijerph21081020)
Supplement: Supplementary file 1 [file ijerph-21-01020-s001.zip › ijerph-3136017-supplementary/IJERPH_Pinto_Supp. Mat. 2.pdf]

Supplementary Material 2: raw data for meta-analysis

Table S2.1 - Brain malignant tumors: raw data extracted from eligible papers (for the meta-analysis).

#	Study (treatment/sham comparison)	incidence in exposed	number of exposed animals	incidence in sham	number of sham animals	SAR	Species	Agent
1	Adey1999	4	60	9	60	1-1.6	Rats	ENU
2	Adey2000	14	90	17	90	0.74-1.60	Rats	ENU
3	Heikkinen2006 (1)	0	72	1	72	0.4	Rats	MX
4	Heikkinen2006 (2)	0	72	1	72	0.9	Rats	MX
5	Heikkinen2001 (1)	1	50	0	49	1.5	Mice	RX
6	Heikkinen2001 (2)	0	50	0	49	0.35	Mice	RX
7	Lerchl2015 (1)	0	92	1	96	0.04	Mice	ENU
8	Lerchl2015 (2)	2	96	1	96	0.4	Mice	ENU
9	Lerchl2015 (3)	1	96	1	96	2	Mice	ENU
10	Shirai2005 (1)	24	100	23	100	0.67	Rats	ENU
11	Shirai2005 (2)	18	100	23	100	2	Rats	ENU
12	Shirai2007 (1)	12	100	9	100	0.67	Rats	ENU
13	Shirai2007 (2)	19	100	9	100	2	Rats	ENU
14	Tilmann2010	0	58	4	60	2.1-5.5	Mice	ENU
15	Zook2001 (1)	36	60	35	60	1	Rats	ENU
16	Zook2001 (2)	7	60	9	58	1	Rats	ENU
17	Zook2001 (3)	9	60	10	60	1	Rats	ENU
18	Zook2001 (4)	3	60	6	60	1	Rats	ENU
19	Zook2002 (1)	60	180	72	180	1	Rats	ENU
20	Zook2002 (2)	95	180	90	180	1	Rats	ENU
21	Zook2006	173	360	193	360	1	Rats	ENU

Table S2.2 - Brain benign tumors: raw data extracted from eligible papers (for the meta-analysis).

#	Study (treatment/sham comparison)	incidence in exposed	number of exposed animals	incidence in sham	number of sham animals	SAR	Species	Agent
1	Heikkinen2006 (1)	0	72	0	72	0.4	Rats	MX
2	Heikkinen2006 (2)	2	72	0	72	0.9	Rats	MX
3	Lerchl2015 (1)	0	92	3	96	0.04	Mice	ENU
4	Lerchl2015 (2)	2	96	3	96	0.4	Mice	ENU
5	Lerchl2015 (3)	0	96	3	96	2	Mice	ENU
6	Shirai2005 (1)	0	100	4	100	0.67	Rats	ENU
7	Shirai2005 (2)	1	100	4	100	2	Rats	ENU
8	Shirai2007 (1)	1	100	0	100	0.67	Rats	ENU
9	Shirai2007 (2)	0	100	0	100	2	Rats	ENU
10	Tilmann2010	2	58	3	60	2.1-5.5	Mice	ENU

Table S2.3 - Breast malignant tumors: raw data extracted from eligible papers (for the meta-analysis).

#	Study (treatment/sham comparison)	incidence in exposed	number of exposed animals	incidence in sham	number of sham animals	SAR	Species	Agent
1	Anane2003 (1)	12	16	10	16	1.4	Rats	DMBA
2	Anane2003 (2)	13	16	10	16	2.2	Rats	DMBA
3	Anane2003 (3)	11	16	10	16	3.5	Rats	DMBA
4	Anane2003 (4)	9	16	9	16	0.1	Rats	DMBA
5	Anane2003 (5)	8	16	9	16	0.7	Rats	DMBA
6	Anane2003 (6)	3	16	9	16	1.4	Rats	DMBA
7	Bartsch2002 (1)	16	20	16	20	0.0175-0.07	Rats	DMBA
8	Bartsch2002 (2)	19	20	17	20	0.0175-0.07	Rats	DMBA
9	Bartsch2002 (3)	16	20	18	20	0.0175-0.07	Rats	DMBA
10	Heikkinen2006 (1)	17	72	9	72	0.4	Rats	MX
11	Heikkinen2006 (2)	6	72	9	72	0.9	Rats	MX
12	Hruby2008 (1)	40	100	30	100	0.4	Rats	DMBA
13	Hruby2008 (2)	35	100	30	100	1.3	Rats	DMBA
14	Hruby2008 (3)	47	100	30	100	4	Rats	DMBA
15	Yu2006 (1)	25	100	37	100	0.44	Rats	DMBA
16	Yu2006 (2)	34	99	37	100	1.33	Rats	DMBA
17	Yu2006 (3)	38	100	37	100	4	Rats	DMBA
18	Zook2001 (4)	3	60	6	60	1	Rats	ENU
19	Zook2002 (1)	60	180	72	180	1	Rats	ENU
20	Zook2002 (2)	95	180	90	180	1	Rats	ENU
21	Zook2006	173	360	193	360	1	Rats	ENU

Table S2.4 - Breast benign tumors: raw data extracted from eligible papers (for the meta-analysis).

#	Study (treatment/sham comparison)	incidence in exposed	number of exposed animals	incidence in sham	number of sham animals	SAR	Species	Agent
1	Bartsch2002 (1)	18	20	18	20	0.0175-0.07	Rats	DMBA
2	Bartsch2002 (2)	12	20	8	20	0.0175-0.07	Rats	DMBA
3	Bartsch2002 (3)	18	20	18	20	0.0175-0.07	Rats	DMBA
4	Heikkinen2006 (1)	31	72	29	72	0.4	Rats	MX
5	Heikkinen2006 (2)	29	72	29	72	0.9	Rats	MX
6	Hruby2008 (1)	17	100	30	100	0.4	Rats	DMBA
7	Hruby2008 (2)	15	100	30	100	1.3	Rats	DMBA
8	Hruby2008 (3)	18	100	30	100	4	Rats	DMBA

Table S2.5 - Skin malignant tumors: raw data extracted from eligible papers (for the meta-analysis).

#	Study (treatment/sham comparison)	incidence in exposed	number of exposed animals	incidence in sham	number of sham animals	SAR	Species	Agent
1	Heikkinen2006 (1)	1	72	0	72	0.4	Rats	MX
2	Heikkinen2006 (2)	2	72	0	72	0.9	Rats	MX
3	Heikkinen2003 (1)	8	20	6	19	0.5	Mice	UV
4	Heikkinen2003 (2)	4	26	2	26	0.5	Mice	UV
5	Heikkinen2003 (3)	6	21	6	19	0.5	Mice	UV
6	Heikkinen2003 (4)	5	27	2	26	0.5	Mice	UV
7	Heikkinen2001 (1)	1	50	0	50	1.5	Mice	RX
8	Heikkinen2001 (2)	0	50	0	50	0.35	Mice	RX
9	Szmigielski1982 (2)	32	40	21	40	2-3	Mice	BaP
10	Szmigielski1982 (3)	38	40	21	40	6-8	Mice	BaP
11	Szudzinski1982 (1)	80	100	84	100	2-3	Mice	BaP
12	Szudzinski1982 (2)	90	100	84	100	6-8	Mice	BaP
13	Yu2006 (1)	0	100	1	100	0.44	Rats	DMBA
14	Yu2006 (2)	0	99	1	100	1.33	Rats	DMBA
15	Yu2006 (3)	2	100	1	100	4	Rats	DMBA

Table S2.6 - Skin benign tumors: raw data extracted from eligible papers (for the meta-analysis).

#	Study (treatment/sham comparison)	incidence in exposed	number of exposed animals	incidence in sham	number of sham animals	SAR	Species	Agent
1	Heikkinen2001 (1)	1	50	0	50	1.5	Mice	RX
2	Heikkinen2001 (2)	0	50	0	50	0.35	Mice	RX
3	Heikkinen2006 (1)	2	72	5	72	0.4	Rats	MX
4	Heikkinen2006 (2)	3	72	5	72	0.9	Rats	MX
5	Heikkinen2003 (1)	0	20	0	19	0.5	Mice	UV
6	Heikkinen2003 (2)	1	26	1	26	0.5	Mice	UV
7	Heikkinen2003 (3)	0	21	0	19	0.5	Mice	UV
8	Heikkinen2003 (4)	0	27	1	26	0.5	Mice	UV

Table S2.7 – Histiocytic Sarcoma: raw data extracted from eligible papers (for the meta-analysis).

#	Study (treatment/sham comparison)	incidence in exposed	number of exposed animals	incidence in sham	number of sham animals	SAR	Species	Agent
1	Heikkinen 2001 (1)	4	50	2	50	1.5	Rats	DMBA
2	Heikkinen 2001 (2)	3	50	2	50	0.35	Rats	DMBA
3	Heikkinen 2006 (1)	1	72	1	72	0.3	Rats	DMBA
4	Heikkinen 2006 (2)	1	72	1	72	0.9	Rats	DMBA
5	Lerchl2015 (1)	2	93	4	96	0.04	Mice	ENU
6	Lerchl2015 (2)	1	96	4	96	0.4	Mice	ENU
7	Lerchl2015 (3)	2	96	4	96	2	Mice	ENU
8	Tilmann2010	1	58	3	60	2.1-5.5	Mice	ENU

Table S2.8 – Kidney malignant tumors: raw data extracted from eligible papers (for the meta-analysis).

#	*Study (treatment/sham comparison)*	*incidence in exposed*	*number of exposed animals*	*incidence in sham*	*number of sham animals*	*SAR*	*Species*	*Agent*
1	Heikkinen 2001 (1)	2	50	0	50	1.5	Rats	DMBA
2	Heikkinen 2001 (2)	0	50	0	50	0.35	Rats	DMBA
3	Heikkinen 2006 (1)	4	72	1	72	0.3	Rats	DMBA
4	Heikkinen 2006 (2)	0	72	1	72	0.9	Rats	DMBA
5	Lerchl2015 (1)	3	91	2	96	0.04	Mice	ENU
6	Lerchl2015 (2)	7	96	2	96	0.4	Mice	ENU
7	Lerchl2015 (3)	5	96	2	96	2	Mice	ENU
8	Tilmann2010	2	58	1	60	2.1-5.5	Mice	ENU

Table S2.9 – Kidney benign tumors: raw data extracted from eligible papers (for the meta-analysis).

#	*Study (treatment/sham comparison)*	*incidence in exposed*	*number of exposed animals*	*incidence in sham*	*number of sham animals*	*SAR*	*Species*	*Agent*
1	Heikkinen 2001 (1)	0	50	1	50	1.5	Rats	DMBA
2	Heikkinen 2001 (2)	2	50	1	50	0.35	Rats	DMBA
3	Heikkinen 2006 (1)	0	72	1	72	0.3	Rats	DMBA
4	Heikkinen 2006 (2)	0	72	1	72	0.9	Rats	DMBA
5	Lerchl2015 (1)	2	91	3	96	0.04	Mice	ENU
6	Lerchl2015 (2)	5	96	3	96	0.4	Mice	ENU
7	Lerchl2015 (3)	2	96	3	96	2	Mice	ENU
8	Tilmann2010	0	58	1	60	2.1-5.5	Mice	ENU

Table S2.10 – Liver malignant tumors: raw data extracted from eligible papers (for the meta-analysis).

#	*Study (treatment/sham comparison)*	*incidence in exposed*	*number of exposed animals*	*incidence in sham*	*number of sham animals*	*SAR*	*Species*	*Agent*
1	Heikkinen 2001 (1)	7	50	4	50	1.5	Rats	DMBA
2	Heikkinen 2001 (2)	4	50	4	50	0.35	Rats	DMBA
3	Heikkinen 2006 (1)	2	72	1	72	0.3	Rats	DMBA
4	Heikkinen 2006 (2)	1	72	1	72	0.9	Rats	DMBA
5	Lerchl2015 (1)	34	93	18	96	0.04	Mice	ENU
6	Lerchl2015 (2)	28	96	18	96	0.4	Mice	ENU
7	Lerchl2015 (3)	32	95	18	96	2	Mice	ENU
8	Tilmann2010	31	58	32	60	2.1-5.5	Mice	ENU

Table S2.11 – Liver benign tumors: raw data extracted from eligible papers (for the meta-analysis).

#	*Study (treatment/sham comparison)*	*incidence in exposed*	*number of exposed animals*	*incidence in sham*	*number of sham animals*	*SAR*	*Species*	*Agent*
1	Heikkinen 2001 (1)	5	50	9	50	1.5	Rats	DMBA
2	Heikkinen 2001 (2)	5	50	9	50	0.35	Rats	DMBA
3	Heikkinen 2006 (1)	6	72	8	72	0.3	Rats	DMBA
4	Heikkinen 2006 (2)	9	72	8	72	0.9	Rats	DMBA
5	Lerchl2015 (1)	38	93	37	96	0.04	Mice	ENU
6	Lerchl2015 (2)	36	96	37	96	0.4	Mice	ENU
7	Lerchl2015 (3)	35	95	37	96	2	Mice	ENU
8	Tilmann2010	49	58	30	60	2.1-5.5	Mice	ENU

Table S2.12 – Lung malignant tumors: raw data extracted from eligible papers (for the meta-analysis).

#	Study (treatment/sham comparison)	incidence in exposed	number of exposed animals	incidence in sham	number of sham animals	SAR	Species	Agent
1	Heikkinen 2001 (1)	1	50	3	50	1.5	Rats	DMBA
2	Heikkinen 2001 (2)	2	50	3	50	0.35	Rats	DMBA
3	Heikkinen 2006 (1)	1	72	0	72	0.3	Rats	DMBA
4	Heikkinen 2006 (2)	0	72	0	72	0.9	Rats	DMBA
5	Lerchl2015 (1)	74	94	81	96	0.04	Mice	ENU
6	Lerchl2015 (2)	92	96	81	96	0.4	Mice	ENU
7	Lerchl2015 (3)	77	95	81	96	2	Mice	ENU
8	Tilmann2010	45	58	33	60	2.1-5.5	Mice	ENU

Table S2.13 – Lung benign tumors: raw data extracted from eligible papers (for the meta-analysis).

#	Study (treatment/sham comparison)	incidence in exposed	number of exposed animals	incidence in sham	number of sham animals	SAR	Species	Agent
1	Heikkinen 2001 (1)	5	50	5	50	1.5	Rats	DMBA
2	Heikkinen 2001 (2)	9	50	5	50	0.35	Rats	DMBA
3	Heikkinen 2006 (1)	0	72	1	72	0.3	Rats	DMBA
4	Heikkinen 2006 (2)	0	72	1	72	0.9	Rats	DMBA
5	Lerchl2015 (1)	43	94	22	96	0.04	Mice	ENU
6	Lerchl2015 (2)	43	96	22	96	0.4	Mice	ENU
7	Lerchl2015 (3)	37	95	22	96	2	Mice	ENU
8	Tilmann2010	36	58	27	60	2.1-5.5	Mice	ENU

Table S2.14 – Lymphoma: raw data extracted from eligible papers (for the meta-analysis).

#	Study (treatment/sham comparison)	incidence in exposed	number of exposed animals	incidence in sham	number of sham animals	SAR	Species	Agent
1	Heikkinen 2001 (1)	12	50	12	50	1.5	Rats	DMBA
2	Heikkinen 2001 (2)	10	50	12	50	0.35	Rats	DMBA
3	Heikkinen 2006 (1)	2	72	1	72	0.3	Rats	DMBA
4	Heikkinen 2006 (2)	0	72	1	72	0.9	Rats	DMBA
5	Lerchl2015 (1)	16	93	9	96	0.04	Mice	ENU
6	Lerchl2015 (2)	23	96	9	96	0.4	Mice	ENU
7	Lerchl2015 (3)	9	96	9	96	2	Mice	ENU
8	Tilmann2010	4	58	4	60	2.1-5.5	Mice	ENU

Table S2.15 – Spleen malignant tumors: raw data extracted from eligible papers (for the meta-analysis).

#	Study (treatment/sham comparison)	incidence in exposed	number of exposed animals	incidence in sham	number of sham animals	SAR	Species	Agent
1	Heikkinen 2001 (1)	2	72	0	72	1.5	Rats	DMBA
2	Heikkinen 2001 (2)	1	72	0	72	0.35	Rats	DMBA
3	Lerchl2015 (1)	1	93	3	96	0.04	Mice	ENU
4	Lerchl2015 (2)	0	96	3	96	0.4	Mice	ENU
5	Lerchl2015 (3)	0	96	3	96	2	Mice	ENU
6	Tilmann2010	0	58	0	60	2.1-5.5	Mice	ENU

Table S2.16 – Spleen benign tumors: raw data extracted from eligible papers (for the meta-analysis).

#	Study (treatment/sham comparison)	incidence in exposed	number of exposed animals	incidence in sham	number of sham animals	SAR	Species	Agent
1	Heikkinen 2001 (1)	0	72	1	72	1.5	Rats	DMBA
2	Heikkinen 2001 (2)	0	72	1	72	0.35	Rats	DMBA
3	Lerchl2015 (1)	1	93	1	96	0.04	Mice	ENU
4	Lerchl2015 (2)	2	96	1	96	0.4	Mice	ENU
5	Lerchl2015 (3)	1	96	1	96	2	Mice	ENU
6	Tilmann2010	1	58	0	60	2.1-5.5	Mice	ENU

Table S2.17 Adrenals malignant tumors: raw data extracted from eligible papers (no meta-analysis).

#	Study (treatment/sham comparison)	incidence in exposed	number of exposed animals	incidence in sham	number of sham animals	SAR	Species	Agent
1	Heikkinen 2001 (1)	0	50	1	50	1.5	Mice	RX
2	Heikkinen 2001 (1)	0	50	1	50	0.35	Mice	RX

Table S2.18 Adrenals benign tumors: raw data extracted from eligible papers (no meta-analysis).

#	Study (treatment/sham comparison)	incidence in exposed	number of exposed animals	incidence in sham	number of sham animals	SAR	Species	Agent
1	Heikkinen 2001 (1)	6	50	12	50	1.5	Mice	RX
2	Heikkinen 2001 (1)	6	50	12	50	0.35	Mice	RX

Table S2.19 Female genital system malignant tumors: raw data extracted from eligible papers (no meta-analysis).

#	Study (treatment/sham comparison)	incidence in exposed	number of exposed animals	incidence in sham	number of sham animals	SAR	Species	Agent
1	Heikkinen 2001 (1)	1	50	0	50	1.5	Mice	RX
2	Heikkinen 2001 (2)	4	50	0	50	0.35	Mice	RX
3	Heikkinen 2006 (1)	4	72	4	72	0.3	Rats	MX
4	Heikkinen 2006 (2)	5	72	4	72	0.9	Rats	MX

Table S2.20 Female genital system benign tumors: raw data extracted from eligible papers (for the meta-analysis).

#	***Study (treatment/sham comparison)***	***incidence in exposed***	***number of exposed animals***	***incidence in sham***	***number of sham animals***	***SAR***	***Species***	***Agent***
1	Heikkinen 2001 (1)	24	50	24	50	1.5	Mice	RX
2	Heikkinen 2001 (2)	24	50	24	50	0.35	Mice	RX
3	Heikkinen 2006 (1)	3	72	4	72	0.3	Rats	MX
4	Heikkinen 2006 (2)	5	72	4	72	0.9	Rats	MX

Table S2.21 Heart malignant tumors: raw data extracted from eligible papers (for the meta-analysis).

#	***Study (treatment/sham comparison)***	***incidence in exposed***	***number of exposed animals***	***incidence in sham***	***number of sham animals***	***SAR***	***Species***	***Agent***
1	Heikkinen 2006 (1)	0	72	0	72	0.3	Rats	MX
2	Heikkinen 2006 (2)	1	72	0	72	0.9	Rats	MX

Table S2.22 Heart benign tumors: raw data extracted from eligible papers (for the meta-analysis).

#	***Study (treatment/sham comparison)***	***incidence in exposed***	***number of exposed animals***	***incidence in sham***	***number of sham animals***	***SAR***	***Species***	***Agent***
1	Heikkinen 2006 (1)	2	72	0	72	0.3	Rats	MX
2	Heikkinen 2006 (2)	0	72	0	72	0.9	Rats	MX

Table S2.23 Hypophysis malignant tumors: raw data extracted from eligible papers (for the meta-analysis).

#	***Study (treatment/sham comparison)***	***incidence in exposed***	***number of exposed animals***	***incidence in sham***	***number of sham animals***	***SAR***	***Species***	***Agent***
1	Heikkinen 2001 (1)	0	50	0	50	1.5	Mice	RX
2	Heikkinen 2001 (2)	1	49	0	49	0.35	Mice	RX

Table S2. 24 Hypophysis benign tumors: raw data extracted from eligible papers (for the meta-analysis).

#	***Study (treatment/sham comparison)***	***incidence in exposed***	***number of exposed animals***	***incidence in sham***	***number of sham animals***	***SAR***	***Species***	***Agent***
1	Heikkinen 2001 (1)	3	50	1	50	1.5	Mice	RX
2	Heikkinen 2001 (2)	2	49	1	49	0.35	Mice	RX

Table S2.25 Mesenteric Lymphnode malignant tumors: raw data extracted from eligible papers (for the meta-analysis).

#	Study (treatment/sham comparison)	incidence in exposed	number of exposed animals	incidence in sham	number of sham animals	SAR	Species	Agent
1	Heikkinen 2006 (1)	0	72	0	72	0.3	Rats	MX
2	Heikkinen 2006 (2)	2	72	0	72	0.9	Rats	MX

Table S2.26 Mesenteric Lymphnode benign tumors: raw data extracted from eligible papers (for the meta-analysis).

#	Study (treatment/sham comparison)	incidence in exposed	number of exposed animals	incidence in sham	number of sham animals	SAR	Species	Agent
1	Heikkinen 2006 (1)	2	72	4	72	0.3	Rats	MX
2	Heikkinen 2006 (2)	9	72	4	72	0.9	Rats	MX

Table S2.27 Pancreas malignant tumors: raw data extracted from eligible papers (for the meta-analysis).

#	Study (treatment/sham comparison)	incidence in exposed	number of exposed animals	incidence in sham	number of sham animals	SAR	Species	Agent
1	Heikkinen 2006 (1)	2	72	3	72	0.3	Rats	MX
2	Heikkinen 2006 (2)	2	72	3	72	0.9	Rats	MX

Table S2.28 Pancreas benign tumors: raw data extracted from eligible papers (for the meta-analysis).

#	Study (treatment/sham comparison)	incidence in exposed	number of exposed animals	incidence in sham	number of sham animals	SAR	Species	Agent
1	Heikkinen 2006 (1)	6	72	4	72	0.3	Rats	MX
2	Heikkinen 2006 (2)	6	72	4	72	0.9	Rats	MX

Table S2.29 Sensor organs (Harderian gland) malignant tumors: raw data extracted from eligible papers (for the meta-analysis).

#	Study (treatment/sham comparison)	incidence in exposed	number of exposed animals	incidence in sham	number of sham animals	SAR	Species	Agent
1	Heikkinen 2001 (1)	1	50	1	50	1.5	Mice	RX
2	Heikkinen 2001 (2)	1	50	1	50	0.35	Mice	RX

Table S2.30 Sensor organs (Harderian gland) benign tumors: raw data extracted from eligible papers (for the meta-analysis).

#	Study (treatment/sham comparison)	incidence in exposed	number of exposed animals	incidence in sham	number of sham animals	SAR	Species	Agent
1	Heikkinen 2001 (1)	30	50	23	50	1.5	Mice	RX
2	Heikkinen 2001 (2)	28	50	23	50	0.35	Mice	RX

Table S2.31 Thymus malignant tumors: raw data extracted from eligible papers (for the meta-analysis).

#	***Study (treatment/sham comparison)***	***incidence in exposed***	***number of exposed animals***	***incidence in sham***	***number of sham animals***	***SAR***	***Species***	***Agent***
1	Heikkinen 2006 (1)	0	69	0	67	0.3	Rats	MX
2	Heikkinen 2006 (2)	1	69	0	67	0.9	Rats	MX

Table S2.32 Thymus benign tumors: raw data extracted from eligible papers (for the meta-analysis).

#	***Study (treatment/sham comparison)***	***incidence in exposed***	***number of exposed animals***	***incidence in sham***	***number of sham animals***	***SAR***	***Species***	***Agent***
1	Heikkinen 2006 (1)	3	69	5	67	0.3	Rats	MX
2	Heikkinen 2006 (2)	3	69	5	67	0.9	Rats	MX

Table S2.33 Thyroid benign tumors: raw data extracted from eligible papers (for the meta-analysis).

#	***Study (treatment/sham comparison)***	***incidence in exposed***	***number of exposed animals***	***incidence in sham***	***number of sham animals***	***SAR***	***Species***	***Agent***
1	Heikkinen 2001 (1)	2	49	0	50	1.5	Mice	RX
2	Heikkinen 2001 (2)	1	50	0	50	0.35	Mice	RX

Table S2.34 Survival: raw data extracted from eligible papers (for the meta-analysis).

#	*Study (treatment/sham comparison)*	*incidence in exposed*	*number of exposed animals*	*incidence in sham*	*number of sham animals*	*SAR*	*Species*	*Agent*
1	Adey1999	44	56	40	60	0	Rats	ENU
2	Adey2000	55	90	56	90	1.17	Rats	ENU
3	Anane2003 (1)	16	16	16	16	1.4	Rats	DMBA
4	Anane2003 (2)	16	16	16	16	2.2	Rats	DMBA
5	Anane2003 (3)	15	16	16	16	3.5	Rats	DMBA
6	Anane2003 (4)	14	16	14	16	0.1	Rats	DMBA
7	Anane2003 (5)	16	16	14	16	0.7	Rats	DMBA
8	Anane2003 (6)	16	16	14	16	1.4	Rats	DMBA
9	Heikkinen2001 (1)	33	50	34	50	1.5	Mice	RX
10	Heikkinen2001 (2)	34	50	34	50	0.35	Mice	RX
11	Heikkinen2003 (1)	19	20	16	19	0.5	Mice	UV
12	Heikkinen2003 (2)	23	26	22	26	0.5	Mice	UV
13	Heikkinen2003 (3)	22	22	16	19	0.5	Mice	UV
14	Heikkinen2003 (4)	23	27	22	26	0.5	Mice	UV
15	Heikkinen2006 (1)	39	72	41	72	0.3	Rats	MX
16	Heikkinen2006 (2)	40	72	41	72	0.9	Rats	MX
17	Hruby2008 (1)	90	100	93	100	0.4	Rats	DMBA
18	Hruby2008 (2)	93	100	93	100	1.3	Rats	DMBA
19	Hruby2008 (3)	89	100	93	100	4	Rats	DMBA
20	Lerchl2015 (1)	23	96	14	96	0.04	Mice	ENU
21	Lerchl2015 (2)	24	96	14	96	0.4	Mice	ENU
22	Lerchl2015 (3)	12	96	14	96	2	Mice	ENU
23	Shirai2005 (1)	53	100	53	100	0.67	Rats	ENU
24	Shirai2005 (2)	63	100	53	100	2	Rats	ENU
25	Shirai2007 (1)	61	100	75	100	0.67	Rats	ENU
26	Shirai2007 (2)	71	100	75	100	2	Rats	ENU
27	Yu2006 (1)	94	100	98	100	0.44	Rats	DMBA
28	Yu2006 (2)	97	100	98	100	1.33	Rats	DMBA
29	Yu2006 (3)	94	100	98	100	4	Rats	DMBA
30	Zook2001 (1)	15	60	19	60	1	Rats	ENU
31	Zook2001 (2)	25	60	25	60	1	Rats	ENU
32	Zook2001 (3)	36	60	35	60	1	Rats	ENU
33	Zook2001 (4)	29	60	35	60	1	Rats	ENU
