# Supplementary material for: A Systematic Review on the In Vivo Studies on Radiofrequency (100 kHz–300 GHz) Electromagnetic Field Exposure and Co-Carcinogenesis
Source: Int J Environ Res Public Health. 2024 Aug 2;21(8):1020. doi: 10.3390/ijerph21081020 (PMC11354106; doi:10.3390/ijerph21081020)
Supplement: Supplementary file 1 [file ijerph-21-01020-s001.zip › ijerph-3136017-supplementary/IJERPH_Pinto_Supp. Mat.3.pdf]

Supplementary Material 3: Meta-analysis results

Table S3.1: Meta-Analysis results for Brain malignant tumors (All Studies)

#	Study (treatment/sham comparison)	Risk Ratio	CI LL	CI UL	Weight (%)
1	Adey1999	0.44	0.14	1.38	0.69%
2	Adey2000	0.82	0.43	1.58	2.09%
3	Heikkinen2006 (1)	0.33	0.01	8.27	0.09%
4	Heikkinen2006 (2)	0.33	0.01	8.27	0.09%
5	Heikkinen2001 (1)	2.94	0.12	73.38	0.09%
6	Heikkinen2001 (2)	0.98	0.02	50.90	0.06%
7	Lerchl2015 (1)	0.35	0.01	8.60	0.09%
8	Lerchl2015 (2)	2.00	0.18	22.02	0.15%
9	Lerchl2015 (3)	1.00	0.06	16.04	0.11%
10	Shirai2005 (1)	1.04	0.63	1.73	3.46%
11	Shirai2005 (2)	0.78	0.45	1.36	2.85%
12	Shirai2007 (1)	1.33	0.59	3.04	1.29%
13	Shirai2007 (2)	2.11	1.00	4.46	1.57%
14	Tilmann2010	0.11	0.01	2.15	0.10%
15	Zook2001 (1)	1.03	0.76	1.39	9.80%
16	Zook2001 (2)	0.75	0.30	1.90	1.02%
17	Zook2001 (3)	0.90	0.39	2.07	1.27%
18	Zook2001 (4)	0.50	0.13	1.93	0.48%
19	Zook2002 (1)	0.83	0.63	1.10	11.59%
20	Zook2002 (2)	1.06	0.86	1.29	21.42%
21	Zook2006	0.90	0.78	1.04	41.70%
	**Combined Effect size**	**0.94**	**0.86**	**1.03**	
	**Heterogeneity**	**$Tau^2 = 0$**	**$I^2 = 0$**		
	**Overall Effect**	**p = 0.136**			

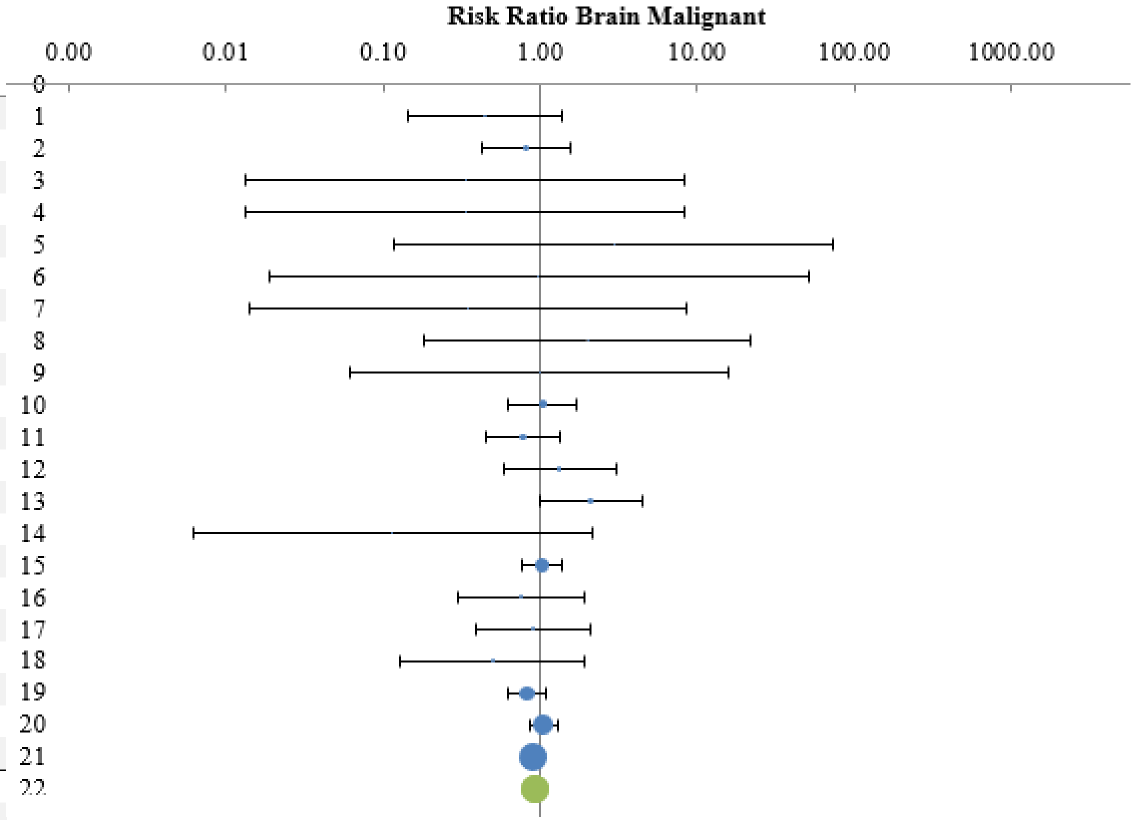

Table S3.2: Subgroup analysis for Brain malignant tumors for the covariate ‘species’

#	Study (treatment/sham comparison)	Risk Ratio	CI LL	CI UL	Weight	p_Q	I^2	T^2
1	Heikkinen2001 (1)	2.94	0.12	73.35	14.37%			
2	Heikkinen2001 (2)	0.98	0.02	50.88	9.53%			
3	Lerchl2015 (1)	0.35	0.01	8.60	14.27%			
4	Lerchl2015 (2)	2.00	0.18	22.02	25.52%			
5	Lerchl2015 (3)	1.00	0.06	16.04	19.07%			
6	Tilmann2010	0.11	0.01	2.15	17.24%			
7	**Mice**	**0.82**	**0.23**	**2.93**	**0.82%**	**0.665**	**0.00%**	**0.00**
8	Adey1999	0.44	0.14	1.38	0.69%			
9	Adey2000	0.82	0.43	1.58	2.10%			
10	Heikkinen2006 (1)	0.33	0.01	8.27	0.09%			
11	Heikkinen2006 (2)	0.33	0.01	8.27	0.09%			
12	Shirai2005 (1)	1.04	0.63	1.73	3.48%			
13	Shirai2005 (2)	0.78	0.45	1.36	2.87%			
14	Shirai2007 (1)	1.33	0.59	3.04	1.30%			
15	Shirai2007 (2)	2.11	1.00	4.46	1.58%			
16	Zook2001 (1)	1.03	0.76	1.39	9.85%			
17	Zook2001 (2)	0.75	0.30	1.90	1.03%			
18	Zook2001 (3)	0.90	0.39	2.07	1.28%			
19	Zook2001 (4)	0.50	0.13	1.93	0.49%			
20	Zook2002 (1)	0.83	0.63	1.10	11.66%			
21	Zook2002 (2)	1.06	0.86	1.29	21.55%			
22	Zook2006	0.90	0.78	1.04	41.95%			
23	**Rats**	**0.94**	**0.85**	**1.03**	**99.18%**	**0.573**	**0.00%**	**0.00**
	**Combined effect size**	**0.94**	**0.92**	**0.96**		**0.735**	**0.00%**	**0.00**

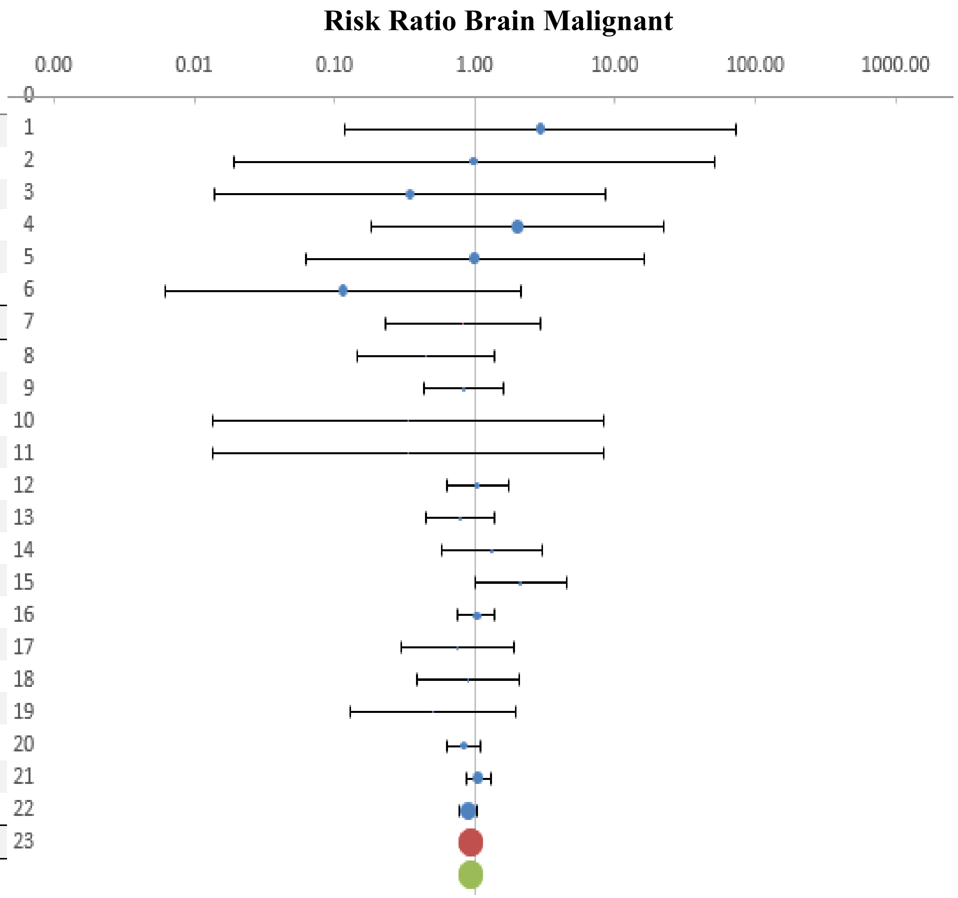

Table S3.3: Meta-Analysis results for Brain malignant tumors (Only ENU treatment)

#	Study (treatment/sham comparison)	Risk Ratio	CI LL	CI UL	Weight (%)
1	Adey1999	0.44	0.14	1.38	0.69%
2	Adey2000	0.82	0.43	1.58	2.09%
3	Lerchl2015 (1)	0.35	0.01	8.60	0.09%
4	Lerchl2015 (2)	2.00	0.18	22.02	0.15%
5	Lerchl2015 (3)	1.00	0.06	16.04	0.11%
6	Shirai2005 (1)	1.04	0.63	1.73	3.47%
7	Shirai2005 (2)	0.78	0.45	1.36	2.86%
8	Shirai2007 (1)	1.33	0.59	3.04	1.30%
9	Shirai2007 (2)	2.11	1.00	4.46	1.57%
10	Tilmann2010	0.11	0.01	2.15	0.10%
11	Zook2001 (1)	1.03	0.76	1.39	9.83%
12	Zook2001 (2)	0.75	0.30	1.90	1.03%
13	Zook2001 (3)	0.90	0.39	2.07	1.27%
14	Zook2001 (4)	0.50	0.13	1.93	0.48%
15	Zook2002 (1)	0.83	0.63	1.10	11.63%
16	Zook2002 (2)	1.06	0.86	1.29	21.48%
17	Zook2006	0.90	0.78	1.04	41.83%
	**Combined Effect size**	**0.94**	**0.85**	**1.03**	
	**Heterogeneity**	**$Tau^2 = 0$**	**$I^2 = 0$**		
	**Overall Effect**	**p = 0.169**			

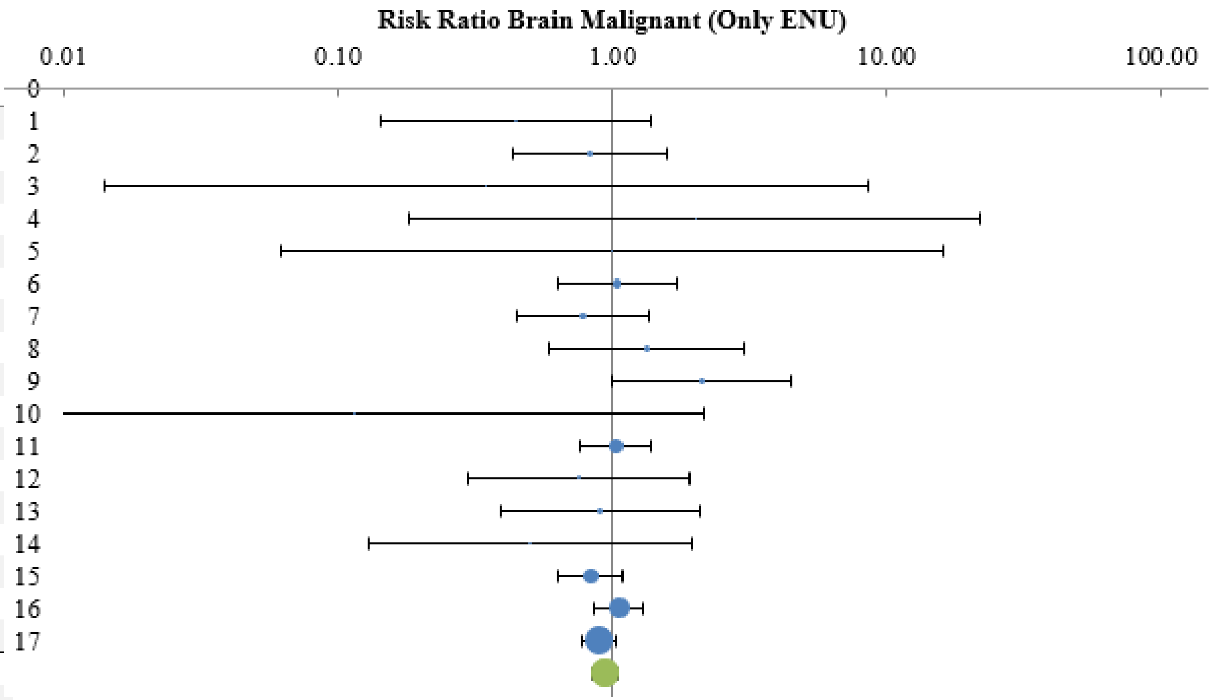

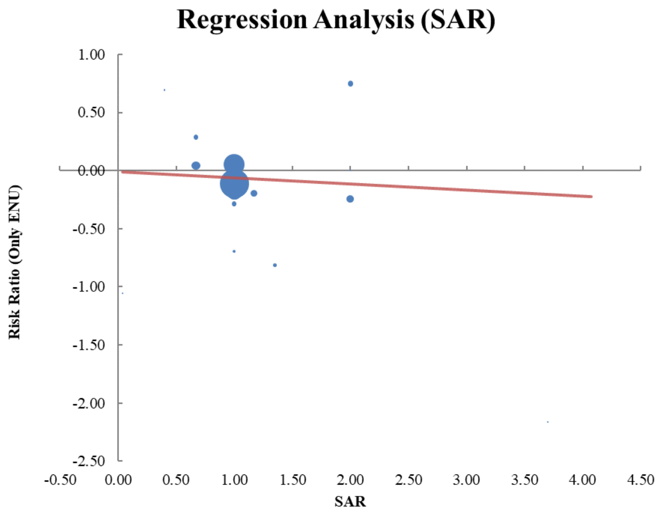

Figure A3.1 Regression Analysis results for the Brain sample (only ENU treatment) according to the covariate ‘SAR’

b = -0.07 **p = 0.786** **R^2 = 0.19**

Table S3.4: Meta-Analysis results for Histiocytic Sarcoma

#	*Study (treatment/sham comparison)*	*Risk Ratio*	*CI LL*	*CI UL*	*Weight (%)*
1	Heikkinen2001 (1)	2.00	0.38	10.65	17.58%
2	Heikkinen2001 (2)	1.50	0.26	8.78	15.74%
3	Heikkinen2006 (1)	1.00	0.06	16.06	6.33%
4	Heikkinen2006 (2)	1.00	0.06	16.06	6.33%
5	Lerchl2015 (1)	0.52	0.10	2.78	17.13%
6	Lerchl2015 (2)	0.25	0.03	2.23	10.16%
7	Lerchl2015 (3)	0.50	0.09	2.69	17.12%
8	Tilmann2010	0.34	0.04	3.30	9.61%
	**Combined Effect size**	**0.75**	**0.40**	**1.40**	
	**Heterogeneity**	**Tau² = 0**	**I² = 0**		
	**Overall Effect**	**p = 0.273**			

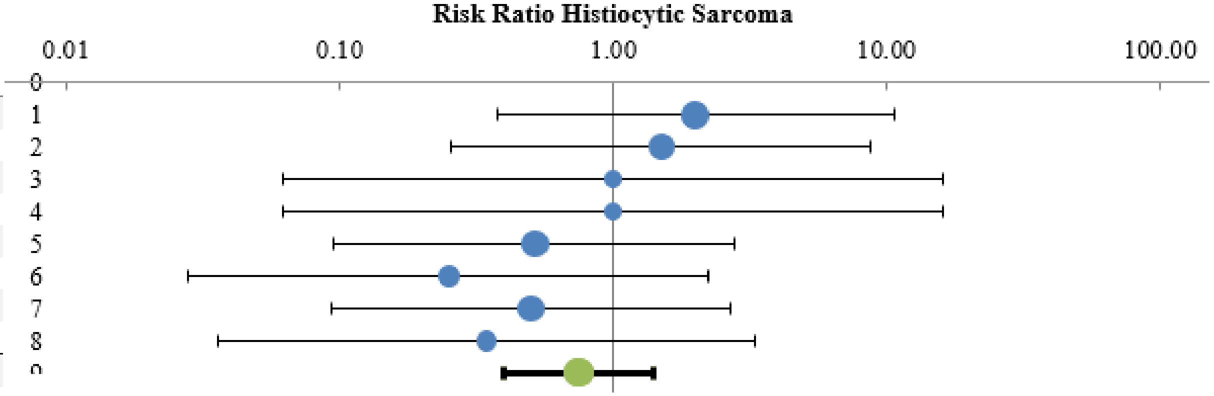

Table S3.5: Subgroup Analysis results for Histiocytic Sarcoma for the covariate 'co-carcinogen agent'

#	*Study (treatment/sham comparison)*	*Risk Ratio*	*CI LL*	*CI UL*	*Weight*	*p_Q*	*I^2*	*T^2*
1	Lerchl2015 (1)	0.52	0.10	2.78	31.71%			
2	Lerchl2015 (2)	0.25	0.03	2.23	18.80%			
3	Lerchl2015 (3)	0.50	0.09	2.69	31.70%			
4	Tilmann2010	0.34	0.04	3.30	17.79%			
5	**ENU**	**0.41**	**0.25**	**0.70**	**######**	**0.951**	**0.00%**	**0.00**
6	Heikkinen2006 (1)	1.00	0.06	16.05	50.00%			
7	Heikkinen2006 (2)	1.00	0.06	16.05	50.00%			
8	**MX**	**1.00**	**1.00**	**1.00**	**######**	**1.000**	**0.00%**	**0.00**
9	Heikkinen2001 (1)	2.00	0.38	10.64	52.77%			
10	Heikkinen2001 (2)	1.50	0.26	8.78	47.23%			
11	**RX**	**1.75**	**0.28**	**10.83**	**######**	**0.814**	**0.00%**	**0.00**
	**Combined effect size**	**#DIV/0!**	**#DIV/0!**	**#DIV/0!**		**0.790**	**0.00%**	**0.00**

Note: Subgroup analysis results are not complete due to the whole MX sub-sample presenting the same incidences in the exposed and sham groups. so its variance has 'zero' value

Table S3.6: Meta-Analysis results for Kidney malignant tumors

#	Study (treatment/sham comparison)	Risk Ratio	CI LL	CI UL	Weight (%)
1	Heikkinen 2001 (1)	5.00	0.24	105.48	6.17%
2	Heikkinen 2001 (2)	1.00	0.02	51.91	3.68%
3	Heikkinen 2006 (1)	4.00	0.45	35.58	11.91%
4	Heikkinen 2006 (2)	0.33	0.01	8.27	5.52%
5	Lerchl2015 (1)	1.58	0.27	9.36	17.94%
6	Lerchl2015 (2)	3.50	0.74	16.58	23.41%
7	Lerchl2015 (3)	2.50	0.49	12.70	21.44%
8	Tilmann2010	2.07	0.19	22.77	9.93%
	**Combined Effect size**	**2.34**	**1.35**	**4.03**	
	**Heterogeneity**	**$Tau^2 = 0$**	**$I^2 = 0$**		
	**Overall Effect**	**p = 0.0002**			

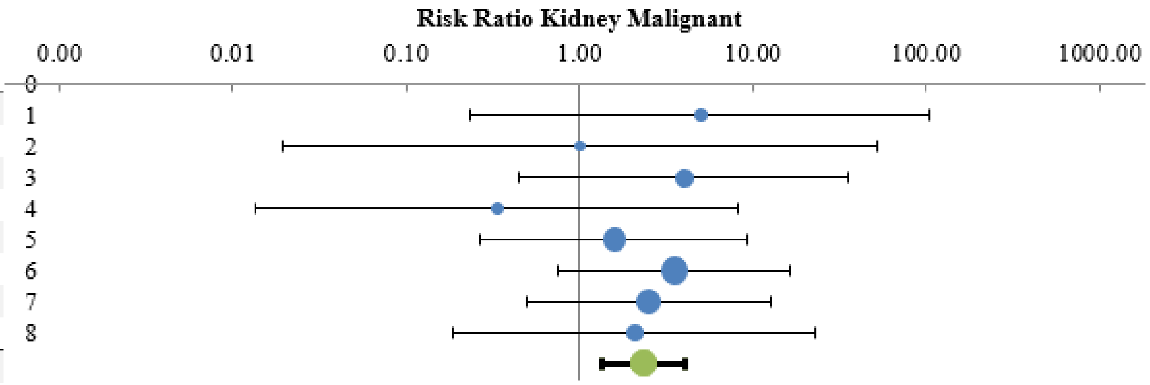

Table S3.7: Subgroup Analysis results for Kidney malignant tumors for the covariate 'co-carcinogen agent'

#	Study (treatment/sham comparison)	Risk Ratio	CI LL	CI UL	Weight	p_Q	I^2	T^2
1	Lerchl2015 (1)	1.58	0.27	9.36	24.66%			
2	Lerchl2015 (2)	3.50	0.74	16.58	32.19%			
3	Lerchl2015 (3)	2.50	0.49	12.70	29.48%			
4	Tilmann2010	2.07	0.19	22.76	13.66%			
5	**ENU**	**2.43**	**1.39**	**4.23**	**93.32%**	**0.928**	**0.00%**	**0.00**
6	Heikkinen 2006 (1)	4.00	0.45	35.57	61.47%			
7	Heikkinen 2006 (2)	0.33	0.01	8.27	38.53%			
8	**MX**	**1.54**	**0.00000033**	**7235442.58**	**1.96%**	**0.206**	**37.46%**	**1.16**
9	Heikkinen 2001 (1)	5.00	0.24	105.44	62.66%			
10	Heikkinen 2001 (2)	1.00	0.02	51.88	37.34%			
11	**RX**	**2.74**	**0.0001386**	**54197.58**	**4.72%**	**0.522**	**0.00%**	**0.00**
	**Combined effect size**	**2.42**	**2.15**	**2.71**		**0.922**	**0.00%**	**0.00**

Table S3.8: Meta-Analysis results for Liver malignant tumors

#	*Study (treatment/sham comparison)*	*Risk Ratio*	*CI LL*	*CI UL*	*Weight (%)*
1	Heikkinen 2001 (1)	1.75	0.54	5.69	3.45%
2	Heikkinen 2001 (2)	1.00	0.26	3.84	2.65%
3	Heikkinen 2006 (1)	2.00	0.18	22.01	0.83%
4	Heikkinen 2006 (2)	1.00	0.06	16.06	0.62%
5	Lerchl2015 (1)	1.95	1.18	3.21	18.63%
6	Lerchl2015 (2)	1.56	0.92	2.63	16.92%
7	Lerchl2015 (3)	1.80	1.08	2.98	18.06%
8	Tilmann2010	1.00	0.71	1.41	38.85%
	**Combined Effect size**	**1.39**	**1.08**	**1.80**	
	**Heterogeneity**	**$Tau^2 = 0.002$**	**$I^2 = 1.69\%$**		
	**Overall Effect**	**p = 0.0024**			

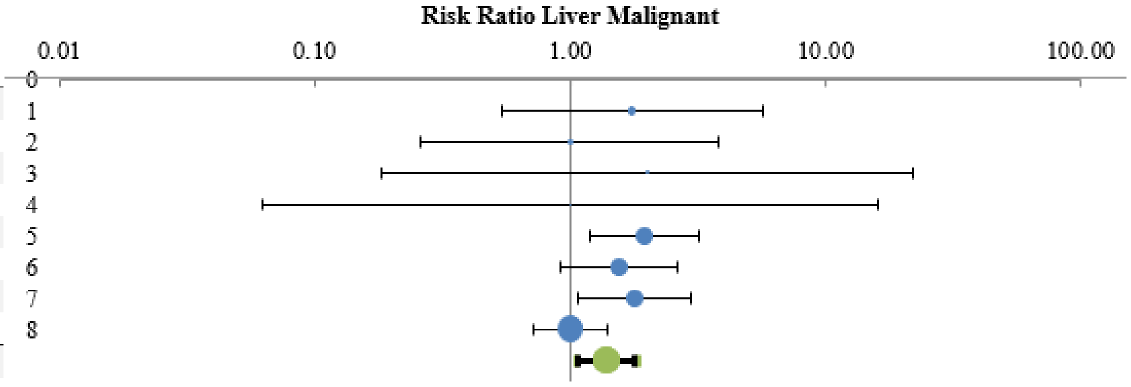

Table S3.9: Subgroup Analysis results for Liver malignant tumors for the covariate 'co-carcinogen agent'

#	*Study (treatment/sham comparison)*	*Risk Ratio*	*CI LL*	*CI UL*	*Weight*	p_Q	I^2	T^2
1	Heikkinen 2001 (1)	0.33	0.03	3.18	29.02%			
2	Heikkinen 2001 (2)	0.67	0.11	3.90	47.31%			
3	Heikkinen 2006 (1)	3.00	0.12	74.43	14.22%			
4	Heikkinen 2006 (2)	1.00	0.02	51.41	9.45%			
5	**DMBA**	**0.70**	**0.20**	**2.49**	**6.70%**	**0.738**	**0.00%**	**0.00**
6	Lerchl2015 (1)	0.93	0.81	1.07	26.67%			
7	Lerchl2015 (2)	1.14	1.03	1.25	30.45%			
8	Lerchl2015 (3)	0.96	0.84	1.09	27.25%			
9	Tilmann2010	1.41	1.08	1.85	15.63%			
10	**ENU**	**1.07**	**0.82**	**1.39**	**93.30%**	**0.008**	**74.92%**	**0.01**
	**Combined effect size**	**1.04**	**0.81**	**1.33**		**0.057**	**48.78%**	**0.01**

Table S3.10: Meta-Analysis results for Lung malignant tumors

#	*Study (treatment/sham comparison)*	*Risk Ratio*	*CI LL*	*CI UL*	*Weight (%)*
1	Heikkinen 2001 (1)	0.33	0.03	3.18	0.32%
2	Heikkinen 2001 (2)	0.67	0.11	3.90	0.52%
3	Heikkinen 2006 (1)	3.00	0.12	74.45	0.16%
4	Heikkinen 2006 (2)	1.00	0.02	51.42	0.10%
5	Lerchl2015 (1)	0.93	0.81	1.07	26.43%
6	Lerchl2015 (2)	1.14	1.03	1.25	31.25%
7	Lerchl2015 (3)	0.96	0.84	1.09	27.15%
8	Tilmann2010	1.41	1.08	1.85	14.07%
	**Combined Effect size**	**1.06**	**0.91**	**1.40**	
	**Heterogeneity**	**Tau² = 0.011**	**I² = 48.78%**		
	**Overall Effect**	**p = 0.375**			

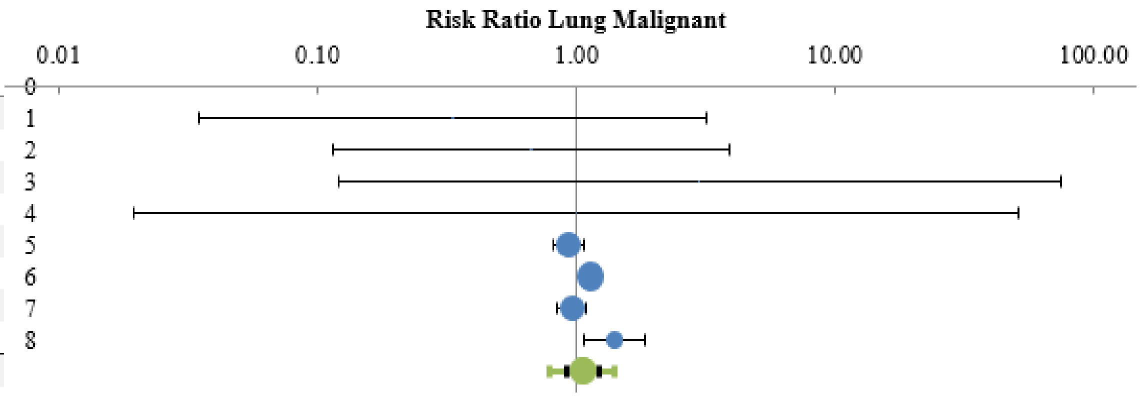

Table S3.11: Subgroup Analysis results for Lung malignant tumors for the covariate 'co-carcinogen agent'

#	*Study (treatment/sham comparison)*	*Risk Ratio*	*CI LL*	*CI UL*	*Weight*	*p_Q*	*I^2*	*T^2*
1	Heikkinen 2001 (1)	0.33	0.03	3.18	29.02%			
2	Heikkinen 2001 (2)	0.67	0.11	3.90	47.31%			
3	Heikkinen 2006 (1)	3.00	0.12	74.43	14.22%			
4	Heikkinen 2006 (2)	1.00	0.02	51.41	9.45%			
5	**DMBA**	**0.70**	**0.20**	**2.49**	**6.70%**	**0.738**	**0.00%**	**0.00**
6	Lerchl2015 (1)	0.93	0.81	1.07	26.67%			
7	Lerchl2015 (2)	1.14	1.03	1.25	30.45%			
8	Lerchl2015 (3)	0.96	0.84	1.09	27.25%			
9	Tilmann2010	1.41	1.08	1.85	15.63%			
10	**ENU**	**1.07**	**0.82**	**1.39**	**93.30%**	**0.008**	**74.92%**	**0.01**
	**Combined effect size**	**1.04**	**0.81**	**1.33**		**0.057**	**48.78%**	**0.01**

Table S3.12: Meta-Analysis results for Lymphoma

#	*Study (treatment/sham comparison)*	*Risk Ratio*	*CI LL*	*CI UL*	*Weight (%)*
1	Heikkinen 2001 (1)	1.00	0.49	2.03	20.92%
2	Heikkinen 2001 (2)	0.83	0.39	1.77	18.69%
3	Heikkinen 2006 (1)	2.00	0.18	22.01	1.97%
4	Heikkinen 2006 (2)	0.33	0.01	8.27	1.10%
5	Lerchl2015 (1)	1.84	0.85	3.96	17.66%
6	Lerchl2015 (2)	2.56	1.24	5.26	19.91%
7	Lerchl2015 (3)	1.00	0.41	2.42	13.63%
8	Tilmann2010	1.03	0.27	4.00	6.10%
	**Combined Effect size**	**1.30**	**0.87**	**1.94**	
	**Heterogeneity**	**$Tau^2 = 0.011$**	**$I^2 = 48.78\%$**		
	**Overall Effect**	**p = 0.119**			

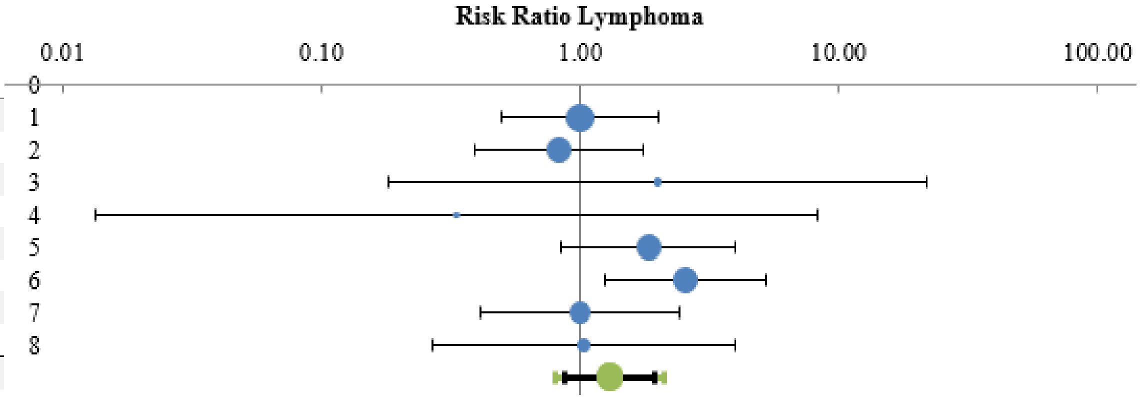

Table S3.13: Subgroup Analysis results for Lymphoma for the covariate ‘co-carcinogen agent’

#	*Study (treatment/sham comparison)*	*Risk Ratio*	*CI LL*	*CI UL*	*Weight*	*p_Q*	*I^2*	*T^2*
1	Heikkinen 2001 (1)	1.00	0.49	2.03	49.56%			
2	Heikkinen 2001 (2)	0.83	0.39	1.77	43.80%			
3	Heikkinen 2006 (1)	2.00	0.18	22.01	4.26%			
4	Heikkinen 2006 (2)	0.33	0.01	8.27	2.38%			
5	**DMBA**	**0.93**	**0.60**	**1.44**	**54.49%**	**0.820**	**0.00%**	**0.00**
6	Lerchl2015 (1)	1.84	0.85	3.96	30.81%			
7	Lerchl2015 (2)	2.56	1.24	5.26	34.72%			
8	Lerchl2015 (3)	1.00	0.41	2.42	23.80%			
9	Tilmann2010	1.03	0.27	4.00	10.67%			
10	**ENU**	**1.68**	**0.82**	**3.44**	**45.51%**	**0.361**	**6.46%**	**0.01**
	**Combined effect size**	**1.21**	**0.60**	**2.44**		**0.389**	**5.40%**	**0.01**

Table S3.14: Meta-Analysis results for Breast malignant tumors

#	Study (treatment/sham comparison)	Risk Ratio	CI LL	CI UL	Weight (%)
1	Anane2003 (1)	1.20	0.73	1.97	4.92%
2	Anane2003 (2)	1.30	0.82	2.07	5.40%
3	Anane2003 (3)	1.10	0.65	1.86	4.46%
4	Anane2003 (4)	1.00	0.53	1.89	3.22%
5	Anane2003 (5)	0.89	0.45	1.76	2.86%
6	Anane2003 (6)	0.33	0.11	1.06	1.08%
7	Bartsch2002 (1)	1.00	0.73	1.38	9.04%
8	Bartsch2002 (2)	1.12	0.90	1.39	13.68%
9	Bartsch2002 (3)	0.89	0.68	1.17	10.94%
10	Heikkinen2006 (1)	1.89	0.90	3.98	2.30%
11	Heikkinen2006 (2)	0.67	0.25	1.79	1.36%
12	Hruby2008 (1)	1.33	0.91	1.96	6.77%
13	Hruby2008 (2)	1.17	0.78	1.75	6.35%
14	Hruby2008 (3)	1.57	1.09	2.26	7.28%
15	Yu2006 (1)	0.68	0.44	1.04	5.82%
16	Yu2006 (2)	0.93	0.64	1.35	7.04%
17	Yu2006 (3)	1.03	0.72	1.47	7.47%
	**Combined Effect size**	**1.06**	**0.93**	**1.21**	
	**Heterogeneity**	**$Tau^2 = 0.015$**	**$I^2 = 26.35\%$**		
	**Overall Effect**	**$p = 0.338$**			

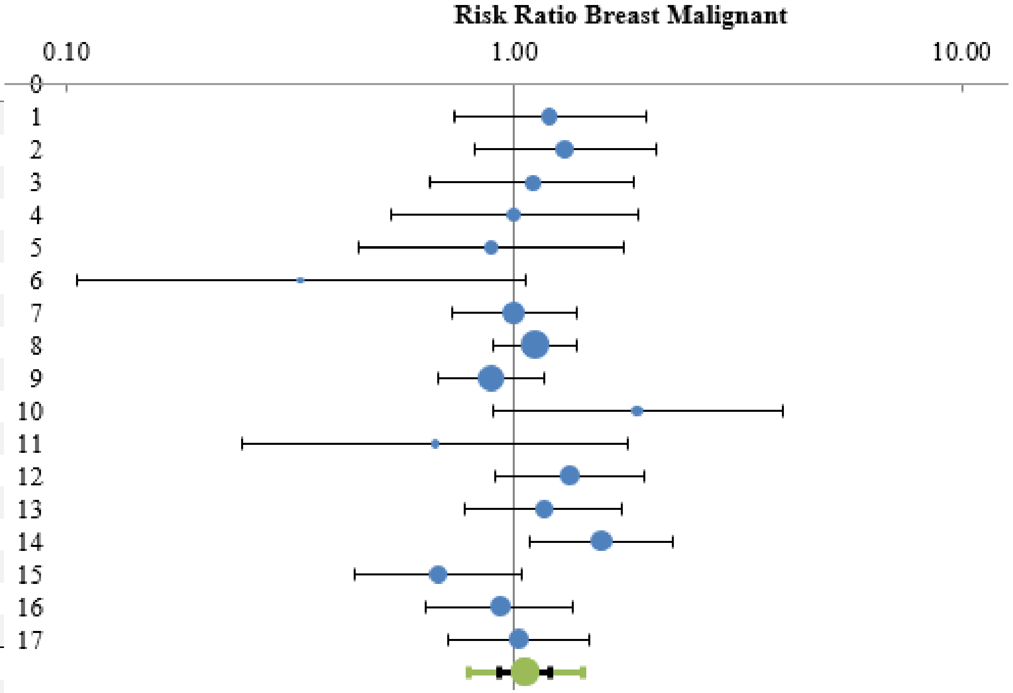

Table S3.15: Meta-Analysis results for Breast malignant tumors (Only DMBA treatment)

#	Study (treatment/sham comparison)	Risk Ratio	CI LL	CI UL	Weight (%)
1	Anane2003 (1)	1.20	0.73	1.97	4.99%
2	Anane2003 (2)	1.30	0.82	2.07	5.49%
3	Anane2003 (3)	1.10	0.65	1.86	4.50%
4	Anane2003 (4)	1.00	0.53	1.89	3.21%
5	Anane2003 (5)	0.89	0.45	1.76	2.85%
6	Anane2003 (6)	0.33	0.11	1.06	1.06%
7	Bartsch2002 (1)	1.00	0.73	1.38	9.46%
8	Bartsch2002 (2)	1.12	0.90	1.39	14.90%
9	Bartsch2002 (3)	0.89	0.68	1.17	11.64%
10	Hruby2008 (1)	1.33	0.91	1.96	6.96%
11	Hruby2008 (2)	1.17	0.78	1.75	6.50%
12	Hruby2008 (3)	1.57	1.09	2.26	7.52%
13	Yu2006 (1)	0.68	0.44	1.04	5.94%
14	Yu2006 (2)	0.93	0.64	1.35	7.25%
15	Yu2006 (3)	1.03	0.72	1.47	7.73%
	**Combined Effect size**	**1.95**	**0.93**	**1.20**	
	**Heterogeneity**	**Tau² = 0.012**	**I² = 24.38**		
	**Overall Effect**	**p = 0.385**			

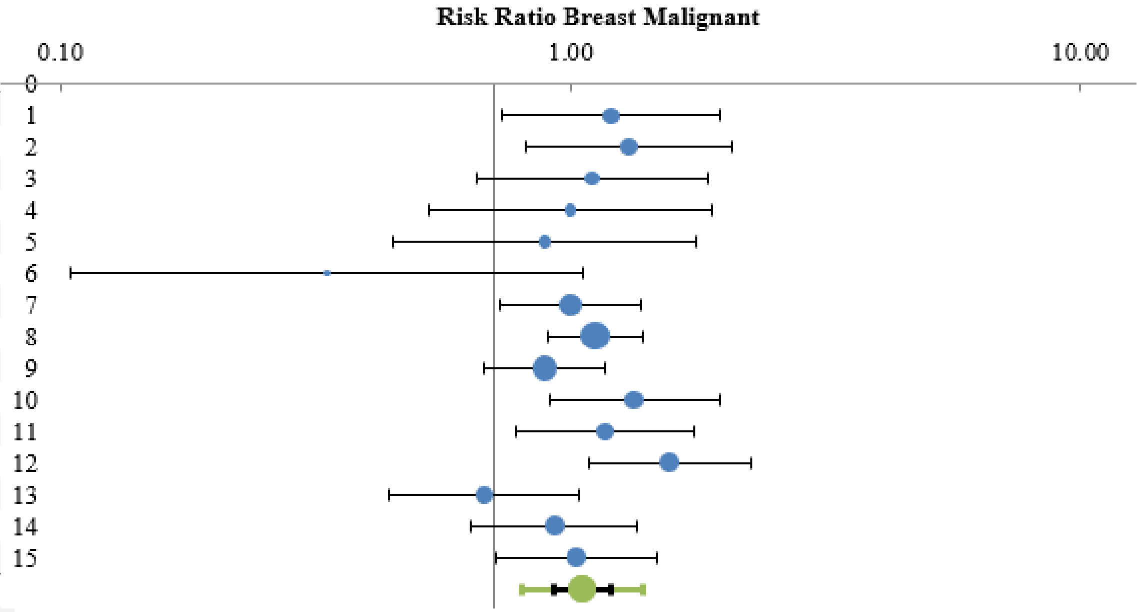

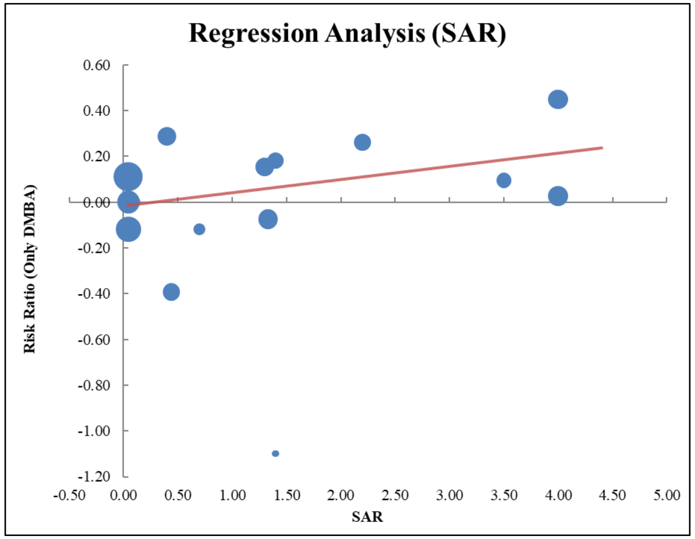

Figure A3.2 Regression Analysis results for the sample Brain (only ENU treatment) according to the covariate 'SAR'

b = 0.37 **p = 0.144** **R^2 = 0.14**

Table S3.16: Meta-Analysis results for Skin malignant tumors

#	*Study (treatment/sham comparison)*	*Risk Ratio*	*CI LL*	*CI UL*	*Weight (%)*
1	Heikkinen2006 (1)	3.00	0.12	74.45	0.30%
2	Heikkinen2006 (2)	5.00	0.24	105.04	0.33%
3	Heikkinen2003 (1)	1.27	0.52	3.06	3.75%
4	Heikkinen2003 (2)	2.00	0.38	10.39	1.15%
5	Heikkinen2003 (3)	0.90	0.34	2.40	3.11%
6	Heikkinen2003 (4)	2.41	0.49	11.76	1.24%
7	Heikkinen2001 (1)	3.00	0.12	74.84	0.30%
8	Heikkinen2001 (2)	1.00	0.02	51.91	0.20%
9	Szmigielski1982 (2)	1.52	1.09	2.14	15.02%
10	Szmigielski1982 (3)	1.81	1.33	2.46	16.56%
11	Szudzinski1982 (1)	0.95	0.84	1.09	27.75%
12	Szudzinski1982 (2)	1.07	0.96	1.19	29.15%
13	Yu2006 (1)	0.33	0.01	8.25	0.30%
14	Yu2006 (2)	0.34	0.01	8.33	0.30%
15	Yu2006 (3)	2.00	0.18	22.03	0.53%
	**Combined Effect size**	**1.22**	**1.03**	**1.45**	
	**Heterogeneity**	**$Tau^2 = 0.024$**	**$I^2 = 41.02\%$**		
	**Overall Effect**	**p = 0.011**			

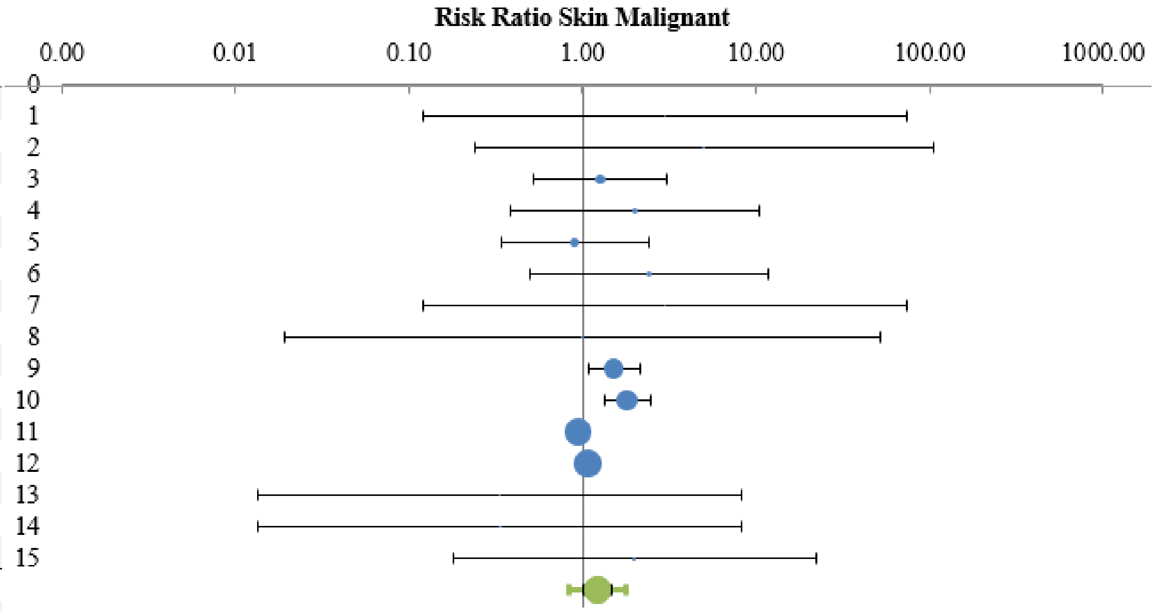

Table S3.17: Subgroup analysis for Skin malignant tumors according to the covariate ‘species’

#	Study name / Subgroup name	Risk Ratio	CI LL	CI UL	Weight	p_Q	I^2	T^2
1	Heikkinen2003 (1)	1.27	0.53	3.05	4.32%			
2	Heikkinen2003 (2)	2.00	0.39	10.38	1.35%			
3	Heikkinen2003 (3)	0.90	0.34	2.40	3.60%			
4	Heikkinen2003 (4)	2.41	0.49	11.76	1.45%			
5	Heikkinen2001 (1)	3.00	0.12	74.81	0.36%			
6	Heikkinen2001 (2)	1.00	0.02	51.88	0.24%			
7	Szmigielski1982 (2)	1.52	1.09	2.14	15.95%			
8	Szmigielski1982 (3)	1.81	1.33	2.46	17.40%			
9	Szudzinski1982 (1)	0.95	0.84	1.09	27.11%			
10	Szudzinski1982 (2)	1.07	0.96	1.19	28.23%			
11	**Mice**	**1.23**	**1.01**	**1.51**	**97.24%**	**0.012**	**57.28%**	**0.03**
12	Heikkinen2006 (1)	3.00	0.12	74.43	16.98%			
13	Heikkinen2006 (2)	5.00	0.24	105.02	18.89%			
14	Yu2006 (1)	0.33	0.01	8.24	16.93%			
15	Yu2006 (2)	0.34	0.01	8.33	16.93%			
16	Yu2006 (3)	2.00	0.18	22.02	30.28%			
17	**Rats**	**1.39**	**0.32**	**6.11**	**2.76%**	**0.638**	**0.00%**	**0.00**
	**Combined effect size**	**1.24**	**1.19**	**1.29**		**0.049**	**41.02%**	**0.02**

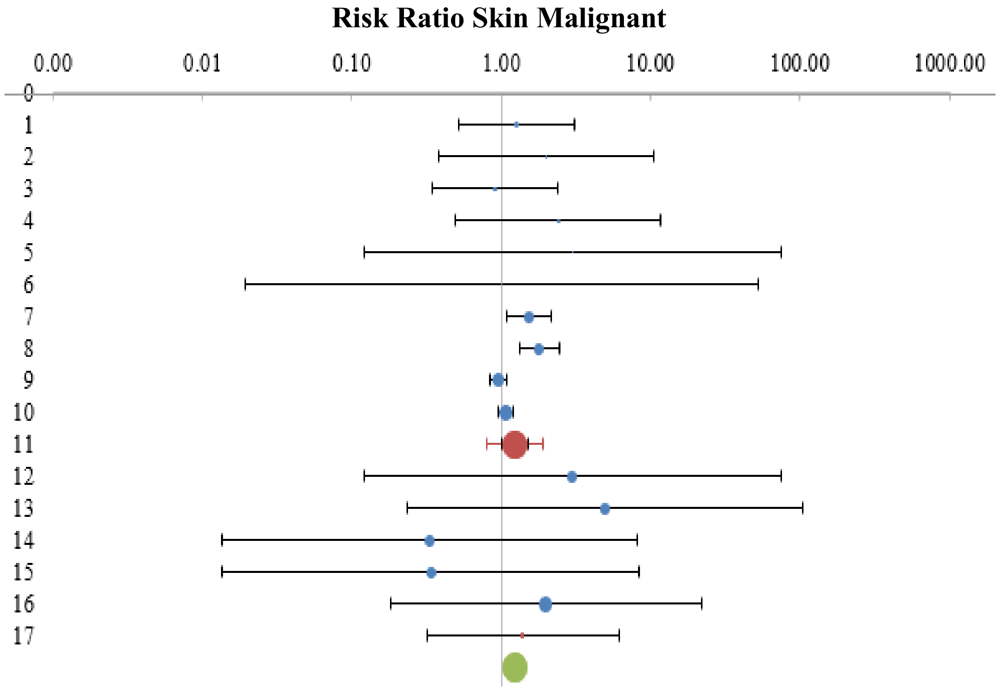

Table S3.18: Subgroup analysis for Skin malignant tumors according to the covariate 'Co-carcinogen agent'

#	Study name / Subgroup name	Risk Ratio	CI LL	CI UL	Weight	p_Q	I^2	T^2
1	Szmigielski1982 (2)	1.52	1.09	2.14	19.33%			
2	Szmigielski1982 (3)	1.81	1.33	2.46	20.77%			
3	Szudzinski1982 (1)	0.95	0.84	1.09	29.50%			
4	Szudzinski1982 (2)	1.07	0.96	1.19	30.40%			
5	**BaP**	**1.24**	**0.77**	**1.97**	**27.26%**	**0.000**	**84.07%**	**0.04**
6	Yu2006 (1)	0.33	0.01	8.24	26.40%			
7	Yu2006 (2)	0.34	0.01	8.33	26.40%			
8	Yu2006 (3)	2.00	0.18	22.02	47.21%			
9	**DMBA**	**0.78**	**0.05**	**11.75**	**10.85%**	**0.566**	**0.00%**	**0.00**
10	Heikkinen2006 (1)	3.00	0.12	74.43	47.34%			
11	Heikkinen2006 (2)	5.00	0.24	105.02	52.66%			
12	**MX**	**3.93**	**0.15**	**100.32**	**23.21%**	**0.820**	**0.00%**	**0.00**
13	Heikkinen2001 (1)	3.00	0.12	74.81	60.12%			
14	Heikkinen2001 (2)	1.00	0.02	51.88	39.88%			
15	**RX**	**1.94**	**0.00**	**1800.18**	**13.13%**	**0.669**	**0.00%**	**0.00**
16	Heikkinen2003 (1)	1.27	0.53	3.05	41.76%			
17	Heikkinen2003 (2)	2.00	0.39	10.38	11.73%			
18	Heikkinen2003 (3)	0.90	0.34	2.40	33.87%			
19	Heikkinen2003 (4)	2.41	0.49	11.76	12.64%			
20	**UV**	**1.29**	**0.69**	**2.41**	**25.56%**	**0.694**	**0.00%**	**0.00**
	**Combined effect size**	**1.65**	**0.94**	**2.90**		**0.049**	**41.02%**	**0.02**

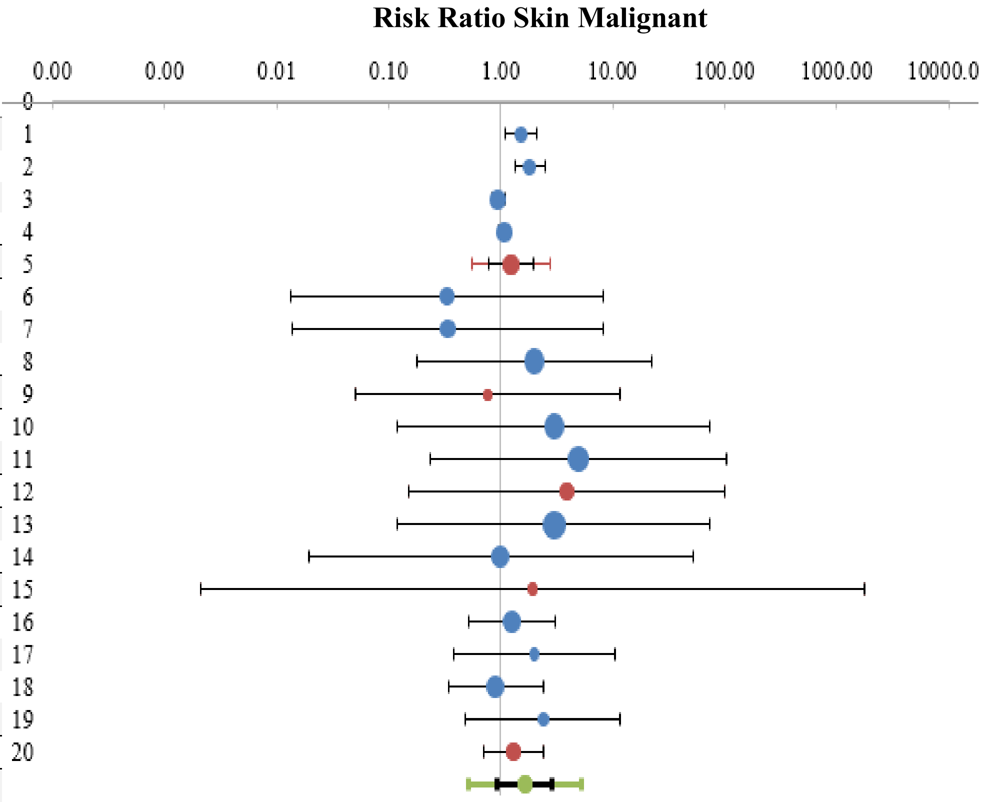

Table S3.19: Meta-Analysis results for Spleen malignant tumors

#	*Study (treatment/sham comparison)*	*Risk Ratio*	*CI LL*	*CI UL*	*Weight (%)*
1	Heikkinen2001 (1)	5.00	0.24	105.04	15.63%
2	Heikkinen2001 (2)	3.00	0.12	74.45	14.05%
3	Lerchl2015 (1)	0.34	0.04	3.30	28.24%
4	Lerchl2015 (2)	0.14	0.01	2.78	16.37%
5	Lerchl2015 (3)	0.14	0.01	2.78	16.37%
6	Tilmann2010	1.03	0.02	53.41	9.35%
	**Combined Effect size**	**0.59**	**0.12**	**2.82**	
	**Heterogeneity**	**Tau² = 0.002**	**I² = 0.08%**		
	**Overall Effect**	**p = 0.385**			

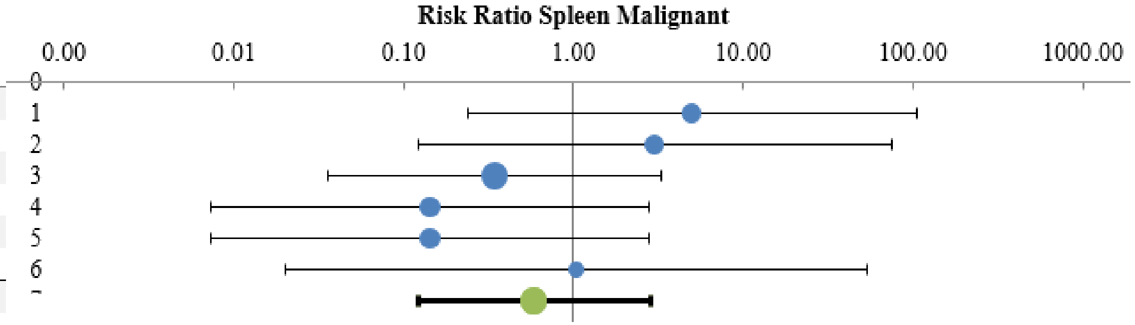

Table S3.20: Subgroup Analysis results for Spleen malignant tumors for the covariate 'co-carcinogen agent'

#	*Study (treatment/sham comparison)*	*Risk Ratio*	*CI LL*	*CI UL*	*Weight*	p_Q	I^2	T^2
1	Lerchl2015 (1)	0.34	0.04	3.30	40.17%			
2	Lerchl2015 (2)	0.14	0.01	2.78	23.27%			
3	Lerchl2015 (3)	0.14	0.01	2.78	23.27%			
4	Tilmann2010	1.03	0.02	53.39	13.29%			
5	**ENU**	**0.26**	**0.08**	**0.91**	**49.41%**	**0.836**	**0.00%**	**0.00**
6	Heikkinen2001 (1)	5.00	0.24	105.02	52.66%			
7	Heikkinen2001 (2)	3.00	0.12	74.43	47.34%			
8	**RX**	**3.93**	**0.15**	**100.32**	**50.59%**	**0.820**	**0.00%**	**0.00**
	**Combined effect size**	**1.04**	**0.03**	**33.16**		**0.415**	**0.08%**	**0.00**

Table S3.21: Meta-Analysis results for Brain benign tumors

#	Study (treatment/sham comparison)	Risk Ratio	CI LL	CI UL	Weight (%)
1	Heikkinen2006 (1)	1.00	0.02	51.42	4.24%
2	Heikkinen2006 (2)	5.00	0.24	105.04	7.10%
3	Lerchl2015 (1)	0.15	0.01	2.90	7.44%
4	Lerchl2015 (2)	0.67	0.11	3.95	20.74%
5	Lerchl2015 (3)	0.14	0.01	2.78	7.44%
6	Shirai2005 (1)	0.11	0.01	2.07	7.65%
7	Shirai2005 (2)	0.25	0.03	2.23	13.70%
8	Shirai2007 (1)	3.00	0.12	74.21	6.37%
9	Shirai2007 (2)	1.00	0.02	51.12	4.23%
10	Tilmann2010	0.69	0.12	4.05	21.08%
	**Combined Effect size**	**0.54**	**0.24**	**1.19**	
	**Heterogeneity**	**$Tau^2 = 0$**	**$I^2 = 0$**		
	**Overall Effect**	**p = 0.078**			

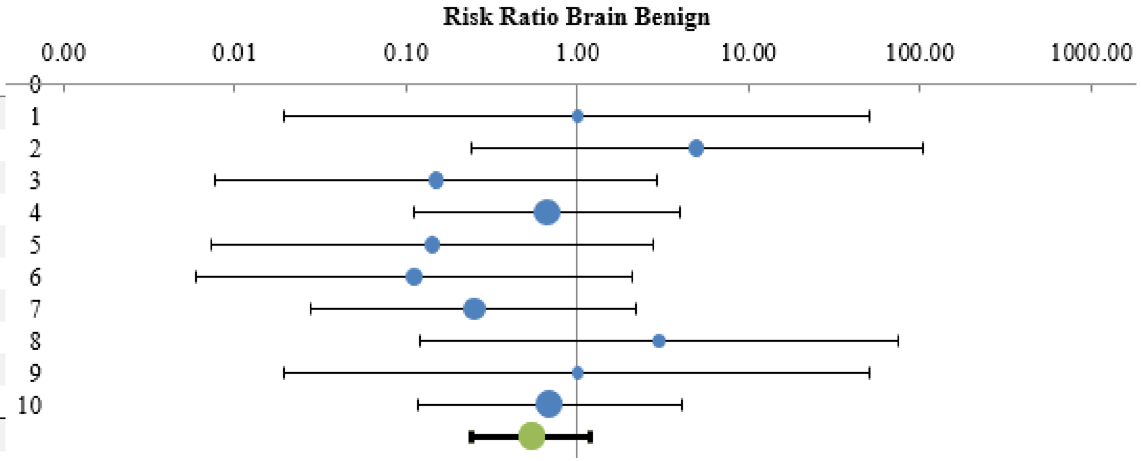

Table S3.22: Subgroup Analysis results for Brain benign tumors for the covariate ‘co-carcinogen agent’

#	Study (treatment/sham comparison)	Risk Ratio	CI LL	CI UL	Weight	p_Q	I^2	T^2
1	Lerchl2015 (1)	0.15	0.01	2.90	8.39%			
2	Lerchl2015 (2)	0.67	0.11	3.95	23.39%			
3	Lerchl2015 (3)	0.14	0.01	2.78	8.39%			
4	Shirai2005 (1)	0.11	0.01	2.07	8.63%			
5	Shirai2005 (2)	0.25	0.03	2.23	15.45%			
6	Shirai2007 (1)	3.00	0.12	74.20	7.18%			
7	Shirai2007 (2)	1.00	0.02	51.12	4.78%			
8	Tilmann2010	0.69	0.12	4.05	23.78%			
9	**ENU**	**0.44**	**0.20**	**0.97**	**57.27%**	**0.754**	**0.00%**	**0.00**
10	Heikkinen2006 (1)	1.00	0.02	51.41	37.39%			
11	Heikkinen2006 (2)	5.00	0.24	105.02	62.61%			
12	**MX**	**2.74**	**0.00**	**54292.97**	**42.73%**	**0.523**	**0.00%**	**0.00**
	**Combined effect size**	**0.96**	**0.12**	**7.48**		**0.674**	**0.00%**	**0.00**

Table S3.23: Meta-Analysis results for Kidney benign tumors

#	*Study (treatment/sham comparison)*	*Risk Ratio*	*CI LL*	*CI UL*	*Weight (%)*
1	Heikkinen 2001 (1)	0.33	0.01	8.32	5.75%
2	Heikkinen 2001 (2)	2.00	0.18	22.00	10.35%
3	Heikkinen 2006 (1)	0.33	0.01	8.27	5.73%
4	Heikkinen 2006 (2)	0.33	0.01	8.27	5.73%
5	Lerchl2015 (1)	0.70	0.12	4.16	18.61%
6	Lerchl2015 (2)	1.67	0.41	6.84	29.49%
7	Lerchl2015 (3)	0.67	0.11	3.95	18.60%
8	Tilmann2010	0.34	0.01	8.57	5.74%
	**Combined Effect size**	**0.84**	**0.47**	**1.51**	
	**Heterogeneity**	**Tau² = 0**	**I² = 0**		
	**Overall Effect**	**p = 0.493**			

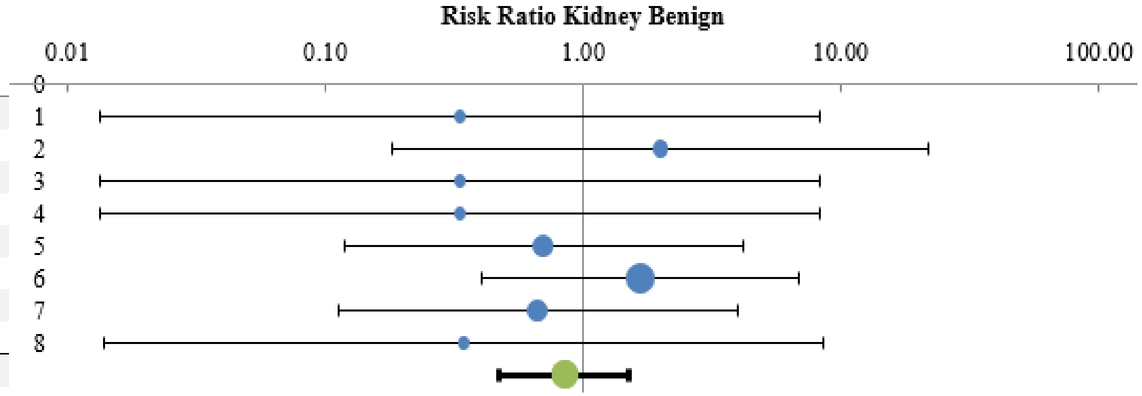

Table S3.24: Subgroup Analysis results for Kidney benign tumors for the covariate ‘co-carcinogen agent’

#	*Study (treatment/sham comparison)*	*Risk Ratio*	*CI LL*	*CI UL*	*Weight*	*p_Q*	*I^2*	*T^2*
1	Heikkinen 2001 (1)	0.33	0.01	8.31	20.87%			
2	Heikkinen 2001 (2)	2.00	0.18	21.99	37.57%			
3	Heikkinen 2006 (1)	0.33	0.01	8.27	20.78%			
4	Heikkinen 2006 (2)	0.33	0.01	8.27	20.78%			
5	**DMBA**	**0.65**	**0.13**	**3.22**	**26.04%**	**0.712**	**0.00%**	**0.00**
6	Lerchl2015 (1)	0.70	0.12	4.16	25.69%			
7	Lerchl2015 (2)	1.67	0.41	6.84	40.71%			
8	Lerchl2015 (3)	0.67	0.11	3.95	25.68%			
9	Tilmann2010	0.34	0.01	8.57	7.92%			
10	**ENU**	**0.93**	**0.36**	**2.40**	**73.96%**	**0.736**	**0.00%**	**0.00**
	**Combined effect size**	**0.85**	**0.59**	**1.23**		**0.902**	**0.00%**	**0.00**

Table S3.25: Meta-Analysis results for Liver benign tumors

#	*Study (treatment/sham comparison)*	*Risk Ratio*	*CI LL*	*CI UL*	*Weight (%)*
1	Heikkinen 2001 (1)	0.56	0.20	1.56	5.19%
2	Heikkinen 2001 (2)	0.56	0.20	1.56	5.19%
3	Heikkinen 2006 (1)	0.75	0.27	2.07	5.31%
4	Heikkinen 2006 (2)	1.13	0.46	2.77	6.42%
5	Lerchl2015 (1)	1.06	0.74	1.51	18.94%
6	Lerchl2015 (2)	0.97	0.68	1.40	18.57%
7	Lerchl2015 (3)	0.96	0.66	1.38	18.43%
8	Tilmann2010	1.69	1.28	2.23	21.94%
	**Combined Effect size**	**1.05**	**0.79**	**1.39**	
	**Heterogeneity**	**Tau² = 0.058**	**I² = 50.27%**		
	**Overall Effect**	**p = 0.714**			

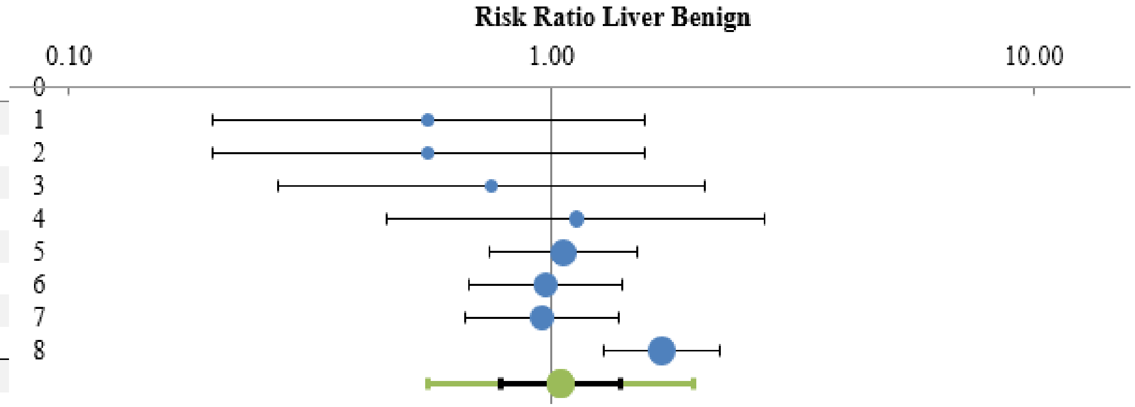

Table S3.26: Subgroup Analysis results for Liver benign tumors for the covariate 'co-carcinogen agent'

#	*Study (treatment/sham comparison)*	*Risk Ratio*	*CI LL*	*CI UL*	*Weight*	p_Q	I^2	T^2
1	Lerchl2015 (1)	1.06	0.74	1.51	24.34%			
2	Lerchl2015 (2)	0.97	0.68	1.40	23.87%			
3	Lerchl2015 (3)	0.96	0.66	1.38	23.70%			
4	Tilmann2010	1.69	1.28	2.23	28.09%			
5	**ENU**	**1.16**	**0.74**	**1.80**	**######**	**0.027**	**67.27%**	**0.06**
6	Heikkinen 2006 (1)	0.75	0.27	2.07	44.12%			
7	Heikkinen 2006 (2)	1.13	0.46	2.77	55.88%			
8	**MX**	**0.94**	**0.07**	**12.15**	**######**	**0.555**	**0.00%**	**0.00**
9	Heikkinen 2001 (1)	0.56	0.20	1.56	50.00%			
10	Heikkinen 2001 (2)	0.56	0.20	1.56	50.00%			
11	**RX**	**0.56**	**0.56**	**0.56**	**######**	**1.000**	**0.00%**	**0.00**
	**Combined effect size**	**#DIV/0!**	**#DIV/0!**	**#DIV/0!**		**0.050**	**50.27%**	**0.06**

Note: Subgroup analysis results are not complete due to the whole RX sub-sample presenting the same incidences in the exposed and sham groups. so its variance has 'zero' value

Table S3.27: Meta-Analysis results for Lung benign tumors

#	*Study (treatment/sham comparison)*	*Risk Ratio*	*CI LL*	*CI UL*	*Weight (%)*
1	Heikkinen 2001 (1)	1.00	0.30	3.29	2.75%
2	Heikkinen 2001 (2)	1.80	0.64	5.06	3.66%
3	Heikkinen 2006 (1)	0.33	0.01	8.27	0.38%
4	Heikkinen 2006 (2)	0.33	0.01	8.27	0.38%
5	Lerchl2015 (1)	2.00	1.30	3.07	20.81%
6	Lerchl2015 (2)	1.95	1.27	3.01	20.71%
7	Lerchl2015 (3)	1.70	1.09	2.66	19.24%
8	Tilmann2010	1.38	0.97	1.95	32.08%
	**Combined Effect size**	**1.65**	**1.35**	**2.01**	
	**Heterogeneity**	**$Tau^2 = 0$**	**$I^2 = 0\%$**		
	**Overall Effect**	**$p \ll 0.01$**			

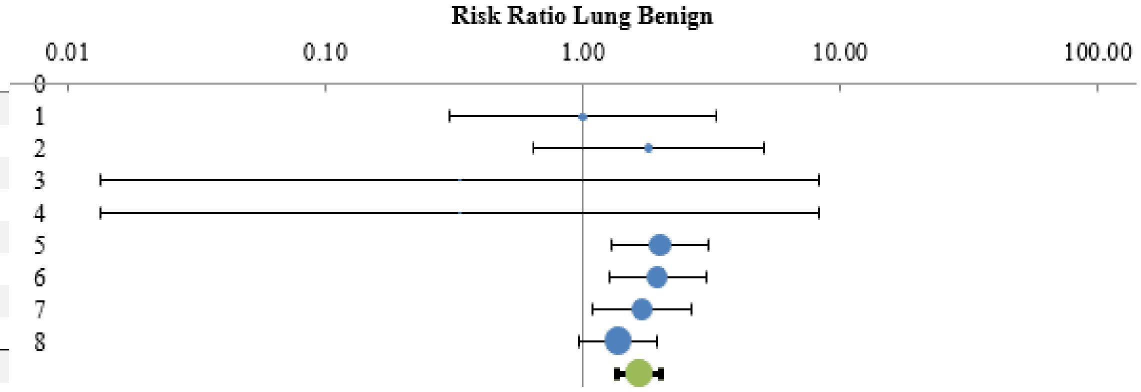

Table S3.28: Subgroup Analysis results for Lung benign tumors for the covariate 'co-carcinogen agent'

#	*Study (treatment/sham comparison)*	*Risk Ratio*	*CI LL*	*CI UL*	*Weight*	p_Q	I^2	T^2
1	Lerchl2015 (1)	2.00	1.30	3.07	22.41%			
2	Lerchl2015 (2)	1.95	1.27	3.01	22.31%			
3	Lerchl2015 (3)	1.70	1.09	2.66	20.72%			
4	Tilmann2010	1.38	0.97	1.95	34.56%			
5	**ENU**	**1.69**	**1.26**	**2.26**	**######**	**0.501**	**0.00%**	**0.00**
6	Heikkinen 2006 (1)	0.33	0.01	8.27	50.00%			
7	Heikkinen 2006 (2)	0.33	0.01	8.27	50.00%			
8	**MX**	**0.33**	**0.33**	**0.33**	**######**	**1.000**	**0.00%**	**0.00**
9	Heikkinen 2001 (1)	1.00	0.30	3.29	42.96%			
10	Heikkinen 2001 (2)	1.80	0.64	5.06	57.04%			
11	**RX**	**1.40**	**0.03**	**56.39**	**######**	**0.459**	**0.00%**	**0.00**
	**Combined effect size**	**#DIV/0!**	**#DIV/0!**	**#DIV/0!**		**0.650**	**0.00%**	**0.00**

Note: Subgroup analysis results are not complete due to the whole MX sub-sample presenting the same incidences in the exposed and sham groups. so its variance has 'zero' value

Table S3.29: Meta-Analysis results for Breast benign tumors

#	*Study (treatment/sham comparison)*	*Risk Ratio*	*CI LL*	*CI UL*	*Weight (%)*
1	Bartsch2002 (1)	1.00	0.81	1.24	20.64%
2	Bartsch2002 (2)	1.50	0.77	2.92	6.70%
3	Bartsch2002 (3)	1.00	0.81	1.24	20.64%
4	Heikkinen2006 (1)	1.07	0.72	1.58	12.93%
5	Heikkinen2006 (2)	1.00	0.67	1.49	12.56%
6	Hruby2008 (1)	0.57	0.33	0.96	8.95%
7	Hruby2008 (2)	0.50	0.29	0.87	8.34%
8	Hruby2008 (3)	0.60	0.36	1.01	9.23%
	**Combined Effect size**	**0.89**	**0.68**	**1.16**	
	**Heterogeneity**	**Tau^2 = 0.036**	**I^2 = 52.30%**		
	**Overall Effect**	**p = 0.291**			

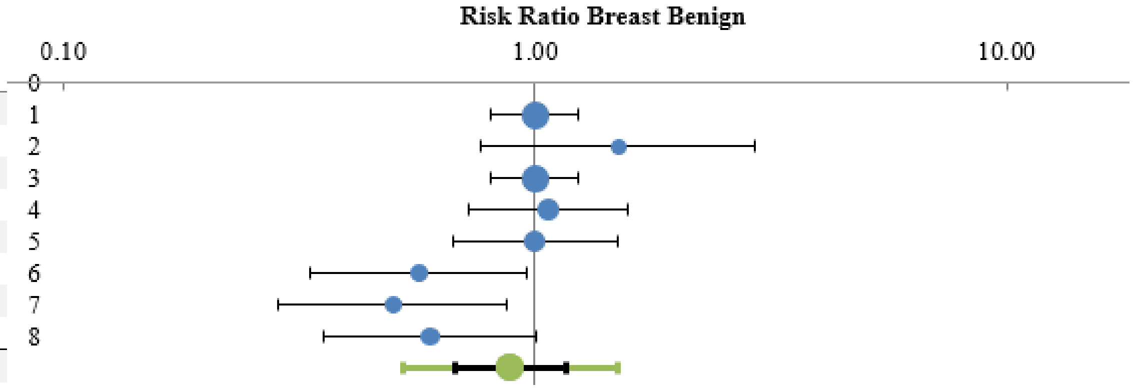

Table S3.30: Subgroup Analysis results for Breast benign tumors for the covariate ‘co-carcinogen agent’

#	*Study (treatment/sham comparison)*	*Risk Ratio*	*CI LL*	*CI UL*	*Weight*	*p_Q*	*I^2*	*T^2*
1	Bartsch2002 (1)	1.00	0.81	1.24	25.10%			
2	Bartsch2002 (2)	1.50	0.77	2.92	10.38%			
3	Bartsch2002 (3)	1.00	0.81	1.24	25.10%			
4	Hruby2008 (1)	0.57	0.33	0.96	13.28%			
5	Hruby2008 (2)	0.50	0.29	0.87	12.52%			
6	Hruby2008 (3)	0.60	0.36	1.01	13.62%			
7	**DMBA**	**0.83**	**0.56**	**1.23**	**27.26%**	**0.016**	**64.30%**	**0.06**
8	Heikkinen2006 (1)	1.07	0.72	1.58	51.39%			
9	Heikkinen2006 (2)	1.00	0.67	1.49	48.61%			
10	**MX**	**1.03**	**0.68**	**1.58**	**72.74%**	**0.814**	**0.00%**	**0.00**
11	**Combined effect size**	**0.97**	**0.77**	**1.23**		**0.040**	**52.30%**	**0.04**

Table S3.31: Meta-Analysis results for Skin benign tumors

#	*Study (treatment/sham comparison)*	*Risk Ratio*	*CI LL*	*CI UL*	*Weight (%)*
1	Heikkinen2001 (1)	3.00	0.12	74.84	6.90%
2	Heikkinen2001 (2)	1.00	0.02	51.91	4.58%
3	Heikkinen2006 (1)	0.40	0.08	2.02	26.97%
4	Heikkinen2006 (2)	0.60	0.15	2.45	35.86%
5	Heikkinen2003 (1)	0.95	0.02	52.14	4.65%
6	Heikkinen2003 (2)	1.00	0.06	16.21	9.43%
7	Heikkinen2003 (3)	0.91	0.02	49.64	4.64%
8	Heikkinen2003 (4)	0.32	0.01	8.15	6.99%
	**Combined Effect size**	**0.64**	**0.39**	**1.05**	
	**Heterogeneity**	**$Tau^2 = 0$**	**$I^2 = 0\%$**		
	**Overall Effect**	**p = 0.033**			

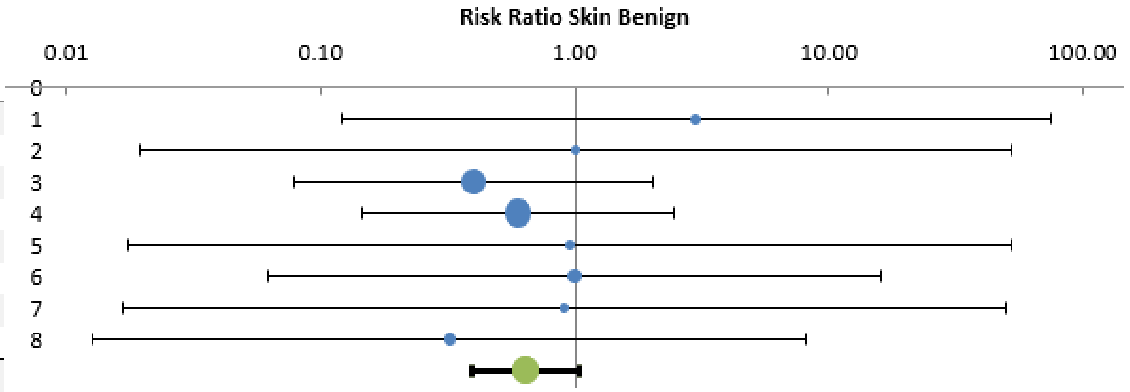

Table S3.32: Subgroup Analysis results for Skin benign tumors for the covariate ‘co-carcinogen agent’

#	*Study (treatment/sham comparison)*	*Risk Ratio*	*CI LL*	*CI UL*	*Weight*	p_Q	I^2	T^2
1	Heikkinen2006 (1)	0.40	0.08	2.02	42.92%			
2	Heikkinen2006 (2)	0.60	0.15	2.45	57.08%			
3	**MX**	**0.50**	**0.04**	**6.46**	**43.30%**	**0.709**	**0.00%**	**0.00**
4	Heikkinen2001 (1)	3.00	0.12	74.81	60.12%			
5	Heikkinen2001 (2)	1.00	0.02	51.88	39.88%			
6	**RX**	**1.94**	**0.00**	**1800.18**	**20.13%**	**0.669**	**0.00%**	**0.00**
7	Heikkinen2003 (1)	0.95	0.02	51.95	18.07%			
8	Heikkinen2003 (2)	1.00	0.06	16.18	36.67%			
9	Heikkinen2003 (3)	0.91	0.02	49.48	18.06%			
10	Heikkinen2003 (4)	0.32	0.01	8.14	27.19%			
11	**UV**	**0.72**	**0.29**	**1.76**	**36.57%**	**0.952**	**0.00%**	**0.00**
	**Combined effect size**	**0.75**	**0.33**	**1.73**		**0.977**	**0.00%**	**0.00**

Table S3.33: Meta-Analysis results for Spleen benign tumors

#	Study (treatment/sham comparison)	Risk Ratio	CI LL	CI UL	Weight (%)
1	Heikkinen2001 (1)	0.33	0.01	8.27	13.42%
2	Heikkinen2001 (2)	0.33	0.01	8.27	13.42%
3	Lerchl2015 (1)	1.03	0.06	16.56	17.89%
4	Lerchl2015 (2)	2.00	0.18	22.02	23.94%
5	Lerchl2015 (3)	1.00	0.06	16.04	17.89%
6	Tilmann2010	3.10	0.12	77.17	13.45%
	**Combined Effect size**	**1.03**	**0.42**	**2.53**	
	**Heterogeneity**	**$Tau^2 = 0$**	**$I^2 = 0\%$**		
	**Overall Effect**	**p = 0.933**			

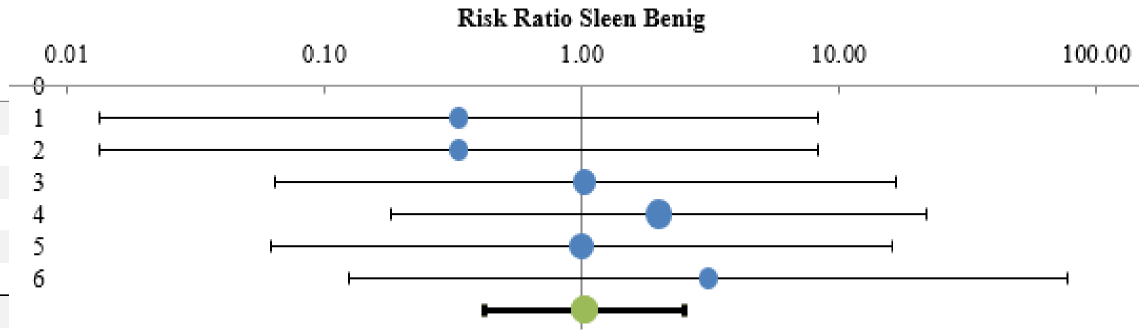

Table S3.34: Subgroup Analysis results for Spleen benign tumors for the covariate 'co-carcinogen agent'

#	Study (treatment/sham comparison)	Risk Ratio	CI LL	CI UL	Weight	p_Q	I^2	T^2
1	Lerchl2015 (1)	1.03	0.06	16.56	24.46%			
2	Lerchl2015 (2)	2.00	0.18	22.02	32.72%			
3	Lerchl2015 (3)	1.00	0.06	16.04	24.45%			
4	Tilmann2010	3.10	0.12	77.15	18.38%			
5	**ENU**	**1.56**	**0.69**	**3.52**	**######**			
6	Heikkinen2001 (1)	0.33	0.01	8.27	50.00%			
7	Heikkinen2001 (2)	0.33	0.01	8.27	50.00%			
8	**RX**	**0.33**	**0.33**	**0.33**	**######**			
	**Combined effect size**	**#DIV/0!**	**#DIV/0!**	**#DIV/0!**				

Note: Subgroup analysis results are not complete due to the whole RX sub-sample presenting the same incidences in the exposed and sham groups. so its variance has 'zero' value
